# Predictions for the leptonic Dirac CP violation phase: a systematic phenomenological analysis

**DOI:** 10.1140/epjc/s10052-015-3559-6

**Published:** 2015-07-24

**Authors:** I. Girardi, S. T. Petcov, A. V. Titov

**Affiliations:** SISSA/INFN, Via Bonomea 265, 34136 Trieste, Italy; Kavli IPMU (WPI), University of Tokyo, 5-1-5 Kashiwanoha, Kashiwa, 277-8583 Japan

## Abstract

We derive predictions for the Dirac phase $\delta$ present in the $3\times 3$ unitary neutrino mixing matrix $U = U_e^{\dagger } \, U_{\nu }$, where $U_e$ and $U_{\nu }$ are $3\times 3$ unitary matrices which arise from the diagonalisation, respectively, of the charged lepton and the neutrino mass matrices. We consider forms of $U_e$ and $U_{\nu }$ allowing us to express $\delta$ as a function of three neutrino mixing angles, present in *U*, and the angles contained in $U_{\nu }$. We consider several forms of $U_{\nu }$ determined by, or associated with, symmetries, tri-bimaximal, bimaximal, etc., for which the angles in $U_{\nu }$ are fixed. For each of these forms and forms of $U_e$ allowing one to reproduce the measured values of the neutrino mixing angles, we construct the likelihood function for $\cos \delta$, using (i) the latest results of the global fit analysis of neutrino oscillation data, and (ii) the prospective sensitivities on the neutrino mixing angles. Our results, in particular, confirm the conclusion, reached in earlier similar studies, that the measurement of the Dirac phase in the neutrino mixing matrix, together with an improvement of the precision on the mixing angles, can provide unique information as regards the possible existence of symmetry in the lepton sector.

## Introduction

Understanding the origin of the observed pattern of neutrino mixing, establishing the status of the CP symmetry in the lepton sector, determining the type of spectrum the neutrino masses obey and determining the nature—Dirac or Majorana—of massive neutrinos are among the highest priority goals of the programme of future research in neutrino physics (see, e.g., [1]). One of the major experimental efforts within this programme will be dedicated to the searches for CP-violating effects in neutrino oscillations (see, e.g., [2–4]). In the reference three neutrino mixing scheme with three light massive neutrinos we are going to consider (see, e.g., [1]), the CP-violating effects in neutrino oscillations can be caused, as is well known, by the Dirac CP violation (CPV) phase present in the Pontecorvo, Maki, Nakagawa, Sakata (PMNS) neutrino mixing matrix. Predictions for the Dirac CPV phase in the lepton sector can be, and were, obtained, in particular, combining the phenomenological approach, developed in [5–10] and further exploited in various versions by many authors with the aim of understanding the pattern of neutrino mixing emerging from the data (see, e.g., [11–21]), with symmetry considerations. In this approach one exploits the fact that the PMNS mixing matrix *U* has the form [8]:

1 $$\begin{aligned} U = U_e^{\dagger }\, U_{\nu } = (\tilde{U}_{e})^\dagger \, \Psi \tilde{U}_{\nu } \, Q_0, \end{aligned}$$

where $U_e$ and $U_{\nu }$ are $3\times 3$ unitary matrices originating from the diagonalisation, respectively, of the charged lepton^1^ and neutrino mass matrices. In Eq. (1) $\tilde{U}_e$ and $\tilde{U}_\nu$ are CKM-like $3\times 3$ unitary matrices, and $\Psi$ and $Q_0$ are diagonal phase matrices each containing in the general case two physical CPV phases^2^:

2 $$\begin{aligned} \Psi = {{\mathrm{diag}}}(1,e^{-i \psi }, e^{-i \omega }), \quad Q_0 = {{{\mathrm{diag}}}} \left( 1,e^{i \frac{\xi _{21}}{2}}, e^{i \frac{\xi _{31}}{2}} \right) .\nonumber \\ \end{aligned}$$

It is further assumed that, up to subleading perturbative corrections (and phase matrices), the PMNS matrix *U* has a specific known form $\tilde{U}_{\nu }$, which is dictated by continuous and/or discrete symmetries, or by arguments related to symmetries. This assumption seems very natural in view of the observation that the measured values of the three neutrino mixing angles differ from certain possible symmetry values by subdominant corrections. Indeed, the best fit values and the 3$\sigma$ allowed ranges of the three neutrino mixing parameters $\sin ^2\theta _{12}$, $\sin ^2\theta _{23}$ and $\sin ^2\theta _{13}$ in the standard parametrisation of the PMNS matrix (see, e.g., [1]), derived in the global analysis of the neutrino oscillation data performed in [23] read

3 $$\begin{aligned}&(\sin ^2 \theta _{12})_\mathrm{BF} = 0.308,\quad 0.259 \le \sin ^2 \theta _{12} \le 0.359,\end{aligned}$$

4 $$\begin{aligned}&(\sin ^2\theta _{23})_\mathrm{BF} = 0.437~(0.455),\nonumber \\&\quad 0.374~(0.380) \le \sin ^2\theta _{23} \le 0.626~(0.641),\end{aligned}$$

5 $$\begin{aligned}&(\sin ^2\theta _{13})_\mathrm{BF} = 0.0234~(0.0240),\nonumber \\&\quad 0.0176~(0.0178) \le \sin ^2\theta _{13} \le 0.0295~(0.0298), \end{aligned}$$

where the value (the value in parentheses) corresponds to $\Delta m^2_{31(32)}>0$ ($\Delta m^2_{31(32)}<0$), i.e., neutrino mass spectrum with normal (inverted) ordering^3^ (see, e.g., [1]). In terms of angles, the best fit values quoted above imply: $\theta _{12} \cong \pi /5.34$, $\theta _{13} \cong \pi /20$ and $\theta _{23} \cong \pi /4.35$. Thus, for instance, $\theta _{12}$ deviates from the possible symmetry value $\pi /4$, corresponding to the bimaximal mixing [25–28], by approximately 0.2, $\theta _{13}$ deviates from 0 (or from 0.32) by approximately 0.16 and $\theta _{23}$ deviates from the symmetry value $\pi /4$ by approximately 0.06, where we used $\sin ^2\theta _{23} = 0.437$.

Widely discussed symmetry forms of $\tilde{U}_{\nu }$ include: (i) tri-bimaximal (TBM) form [7, 29–32], (ii) bimaximal (BM) form, or due to a symmetry corresponding to the conservation of the lepton charge $L' = L_e - L_{\mu } - L_{\tau }$ (LC) [25–28], (iii) golden ratio type A (GRA) form [33, 34], (iv) golden ratio type B (GRB) form [35, 36], and (v) hexagonal (HG) form [21, 37]. For all these forms the matrix $\tilde{U}_{\nu }$ represents a product of two orthogonal matrices describing rotations in the 1–2 and 2–3 planes on fixed angles $\theta ^{\nu }_{12}$ and $\theta ^{\nu }_{23}$:

6 $$\begin{aligned} \tilde{U}_\nu = R_{23}(\theta ^{\nu }_{23}) \, R_{12}(\theta ^{\nu }_{12}), \end{aligned}$$

where

7 $$\begin{aligned} R_{12}\left( \theta ^\nu _{12} \right)= & {} \begin{pmatrix} \cos \theta ^\nu _{12} &{}\quad \sin \theta ^\nu _{12} &{}\quad 0\\ - \sin \theta ^\nu _{12} &{}\quad \cos \theta ^\nu _{12} &{}\quad 0\\ 0 &{}\quad 0 &{}\quad 1 \end{pmatrix} , \quad \nonumber \\ R_{23}\left( \theta ^\nu _{23} \right)= & {} \begin{pmatrix} 1 &{}\quad 0 &{}\quad 0\\ 0 &{}\quad \cos \theta ^\nu _{23} &{}\quad \sin \theta ^\nu _{23} \\ 0 &{}\quad - \sin \theta ^\nu _{23} &{}\quad \cos \theta ^\nu _{23} \\ \end{pmatrix} . \end{aligned}$$

Thus, $\tilde{U}_\nu$ does not include a rotation in the 1–3 plane, i.e., $\theta ^{\nu }_{13} = 0$. Moreover, for all the symmetry forms quoted above one has also $\theta ^{\nu }_{23} = -\,\pi /4$. The forms differ by the value of the angle $\theta ^{\nu }_{12}$, and, correspondingly, of $\sin ^2\theta ^{\nu }_{12}$: for the TBM, BM (LC), GRA, GRB and HG forms we have, respectively, $\sin ^2\theta ^{\nu }_{12} = 1/3$, 1 / 2, $(2 + r)^{-1} \cong 0.276$, $(3 - r)/4 \cong 0.345$, and 1 / 4, *r* being the golden ratio, $r = (1 +\sqrt{5})/2$.

As is clear from the preceding discussion, the values of the angles in the matrix $\tilde{U}_{\nu }$, which are fixed by symmetry arguments, typically differ from the values determined experimentally by relatively small perturbative corrections. In the approach we are following, the requisite corrections are provided by the angles in the matrix $\tilde{U}_e$. The matrix $\tilde{U}_e$ in the general case depends on three angles and one phase [8]. However, in a class of theories of (lepton) flavour and neutrino mass generation, based on a GUT and/or a discrete symmetry (see, e.g., [38–44]), $\tilde{U}_e$ is an orthogonal matrix which describes one rotation in the 1–2 plane,

8 $$\begin{aligned} \tilde{U}_e&= R^{-1}_{12}(\theta ^{e}_{12}), \end{aligned}$$

or two rotations in the planes 1–2 and 2–3,

9 $$\begin{aligned} \tilde{U}_e&= R^{-1}_{23}(\theta ^{e}_{23})\, R^{-1}_{12}(\theta ^{e}_{12}), \end{aligned}$$

$\theta ^{e}_{12}$ and $\theta ^{e}_{23}$ being the corresponding rotation angles. Other possibilities include $\tilde{U}_e$ being an orthogonal matrix which describes (i) one rotation in the 1–3 plane,^4^

10 $$\begin{aligned} \tilde{U}_e&= R^{-1}_{13}(\theta ^{e}_{13}), \end{aligned}$$

or (ii) two rotations in any other two of the three planes, e.g.,

11 $$\begin{aligned} \tilde{U}_e&= R^{-1}_{23}(\theta ^{e}_{23})\, R^{-1}_{13}(\theta ^{e}_{13}),\quad \mathrm{or} \end{aligned}$$

12 $$\begin{aligned} \tilde{U}_e&= R^{-1}_{13}(\theta ^{e}_{13})\, R^{-1}_{12}(\theta ^{e}_{12}). \end{aligned}$$

The use of the inverse matrices in Eqs. (8)–(12) is a matter of convenience—this allows us to lighten the notations in expressions which will appear further in the text.

It was shown in [45] (see also [46]) that for $\tilde{U}_{\nu }$ and $\tilde{U}_e$ given in Eqs. (6) and (9), the Dirac phase $\delta$ present in the PMNS matrix satisfies a sum rule by which it is expressed in terms of the three neutrino mixing angles measured in the neutrino oscillation experiments and the angle $\theta ^{\nu }_{12}$. In the standard parametrisation of the PMNS matrix (see, e.g., [1]) the sum rule reads [45]

13 $$\begin{aligned} \cos \delta= & {} \frac{\tan \theta _{23}}{\sin 2\theta _{12}\sin \theta _{13}} [\cos 2\theta ^{\nu }_{12}+ (\sin ^2\theta _{12} - \cos ^2\theta ^{\nu }_{12}) \nonumber \\&\times (1 - \cot ^2\theta _{23}\,\sin ^2\theta _{13} ) ]. \end{aligned}$$

For the specific values of $\theta ^{\nu }_{12}= \pi /4$ and $\theta ^{\nu }_{12} = \sin ^{-1} (1/\sqrt{3})$, i.e., for the BM (LC) and TBM forms of $\tilde{U}_{\nu }$, Eq. (13) reduces to the expressions for $\cos \delta$ derived first in [46]. On the basis of the analysis performed and the results obtained using the best fit values of $\sin ^2\theta _{12}$, $\sin ^2\theta _{13}$ and $\sin ^2\theta _{23}$, it was concluded in [45], in particular, that the measurement of $\cos \delta$ can allow one to distinguish between the different symmetry forms of the matrix $\tilde{U}_{\nu }$ considered.

Within the approach employed, the expression for $\cos \delta$ given in Eq. (13) is exact. In [45] the correction to the sum rule Eq. (13) due to a non-zero angle $\theta ^{e}_{13} \ll 1$ in $\tilde{U_e}$, corresponding to

14 $$\begin{aligned} \tilde{U}_e = R^{-1}_{23}(\theta ^{e}_{23})\,R^{-1}_{13}(\theta ^{e}_{13})\, R^{-1}_{12}(\theta ^{e}_{12})\, \end{aligned}$$

with $|\sin \theta ^e_{13}| \ll 1$, was also derived.

Using the best fit values of the neutrino mixing parameters $\sin ^2\theta _{12}$, $\sin ^2\theta _{13}$ and $\sin ^2\theta _{23}$, found in the global analysis in [47], predictions for $\cos \delta$, $\delta$ and the rephasing invariant

15 $$\begin{aligned} J_\mathrm{CP}= & {} \mathrm{Im} \{ U^*_{e1} U^*_{\mu 3} U_{e3} U_{\mu 1}\}\nonumber \\= & {} \frac{1}{8} \sin \delta \sin 2\theta _{13} \sin 2\theta _{23} \sin 2\theta _{12} \cos \theta _{13} , \end{aligned}$$

which controls the magnitude of CP-violating effects in neutrino oscillations [48], were presented in [45] for each of the five symmetry forms of $\tilde{U}_{\nu }$—TBM, BM (LC), GRA, GRB and HG—considered.

Statistical analysis of the sum rule Eq. (13) predictions for $\delta$ and $J_\mathrm{CP}$ (for $\cos \delta$) using the current (the prospective) uncertainties in the determination of the three neutrino mixing parameters, $\sin ^2\theta _{12}$, $\sin ^2\theta _{13}$, $\sin ^2\theta _{23}$, and $\delta$ ($\sin ^2\theta _{12}$, $\sin ^2\theta _{13}$ and $\sin ^2\theta _{23}$), was performed in [49, 50] for the five symmetry forms—BM (LC), TBM, GRA, GRB and HG—of $\tilde{U}_{\nu }$. Using the current uncertainties in the measured values of $\sin ^2\theta _{12}$, $\sin ^2\theta _{13}$, $\sin ^2\theta _{23}$ and $\delta$^5^, it was found, in particular, that for the TBM, GRA, GRB and HG forms, $J_\mathrm{CP}\ne 0$ at $5\sigma$, $4\sigma$, $4\sigma$ and $3\sigma$, respectively. For all these four forms $|J_\mathrm{CP}|$ is predicted at $3\sigma$ to lie in the following narrow interval [49, 50]: $0.020 \le |J_\mathrm{CP}| \le 0.039$. As a consequence, in all these cases the CP-violating effects in neutrino oscillations are predicted to be relatively large. In contrast, for the BM (LC) form, the predicted best fit value is $J_\mathrm{CP} \cong 0$, and the CP-violating effects in neutrino oscillations can be strongly suppressed. The statistical analysis of the sum rule predictions for $\cos \delta$, performed in [49, 50] by employing prospective uncertainties of 0.7, 3 and 5 % in the determination of $\sin ^2\theta _{12}$, $\sin ^2\theta _{13}$ and $\sin ^2\theta _{23}$, revealed that with a precision in the measurement of $\delta$, $\Delta \delta \cong (12^\circ \hbox {--}16^\circ )$, which is planned to be achieved in the future neutrino experiments like T2HK and ESS$\nu$SB [4], it will be possible to distinguish at $3\sigma$ between the BM (LC), TBM/GRB and GRA/HG forms of $\tilde{U}_{\nu }$. Distinguishing between the TBM and GRB forms, and between the GRA and HG forms, requires a measurement of $\delta$ with an uncertainty of a few degrees.

In the present article we derive new sum rules for $\cos \delta$ using the general approach employed, in particular, in [45, 49, 50]. We perform a systematic study of the forms of the matrices $\tilde{U}_e$ and $\tilde{U}_\nu$, for which it is possible to derive sum rules for $\cos \delta$ of the type of Eq. (13), but for which the sum rules of interest do not exist in the literature. More specifically, we consider the following forms of $\tilde{U}_e$ and $\tilde{U}_\nu$:

(A) $\tilde{U}_\nu = R_{23}(\theta ^{\nu }_{23})R_{12}(\theta ^{\nu }_{12})$ with $\theta ^{\nu }_{23} = -\pi /4$ and $\theta ^{\nu }_{12}$ corresponding to the TBM, BM (LC), GRA, GRB and HG mixing, and (i) $\tilde{U}_e = R^{-1}_{13}(\theta ^{e}_{13})$, (ii) $\tilde{U}_e = R^{-1}_{23}(\theta ^{e}_{23})R^{-1}_{13}(\theta ^{e}_{13})$, and (iii) $\tilde{U}_e = R^{-1}_{13}(\theta ^{e}_{13}) R^{-1}_{12}(\theta ^{e}_{12})$;
(B) $\tilde{U}_\nu = R_{23}(\theta ^{\nu }_{23})R_{13}(\theta ^{\nu }_{13}) R_{12}(\theta ^{\nu }_{12})$ with $\theta ^{\nu }_{23}$, $\theta ^{\nu }_{13}$ and $\theta ^{\nu }_{12}$ fixed by arguments associated with symmetries, and (iv) $\tilde{U}_e = R^{-1}_{12}(\theta ^{e}_{12})$, and (v) $\tilde{U}_e = R^{-1}_{13}(\theta ^{e}_{13})$.

In each of these cases we obtain the respective sum rule for $\cos \delta$. This is done first for $\theta ^{\nu }_{23} = -\,\pi /4$ in the cases listed in point A, and for the specific values of (some of) the angles in $\tilde{U}_{\nu }$, characterising the cases listed in point B. For each of the cases listed in points A and B we derive also generalised sum rules for $\cos \delta$ for arbitrary fixed values of all angles contained in $\tilde{U}_{\nu }$ (i.e., without setting $\theta ^{\nu }_{23} = -\,\pi /4$ in the cases listed in point A, etc.). Next we derive predictions for $\cos \delta$ and $J_\mathrm{CP}$ ($\cos \delta$), performing a statistical analysis using the current (the prospective) uncertainties in the determination of the neutrino mixing parameters $\sin ^2\theta _{12}$, $\sin ^2\theta _{13}$, $\sin ^2\theta _{23}$ and $\delta$ ($\sin ^2\theta _{12}$, $\sin ^2\theta _{13}$ and $\sin ^2\theta _{23}$).

It should be noted that the approach to understanding the experimentally determined pattern of lepton mixing and to obtaining predictions for $\cos \delta$ and $J_\mathrm{CP}$ employed in the present work and in the earlier related studies [45, 49, 50] is by no means unique—it is one of a number of approaches discussed in the literature on the problem (see, e.g., [51–54]). It is used in a large number of phenomenological studies (see, e.g., [5, 6, 8, 10, 16–20, 55]) as well as in a class of models (see [38–44, 56]) of neutrino mixing based on discrete symmetries. However, it should be clear that the conditions of the validity of the approach employed in the present work are not fulfilled in all theories with discrete flavour symmetries. For example, they are not fulfilled in the theories with discrete flavour symmetry $\Delta (6n^2)$ studied in [57, 58], with the $S_4$ flavour symmetry constructed in [59] and in the models discussed in [60–62]. Further, the conditions of our analysis are also not fulfilled in the phenomenological approach developed and exploited in [52–54]. In these articles, in particular, the matrices $U_e$ and $U_{\nu }$ are assumed to have specific given fixed forms, in which all three mixing angles in each of the two matrices are fixed to some numerical values, typically, but not only, $\pi /4$, or some integer powers *n* of the parameter $\epsilon \cong \theta _C$, $\theta _C$ being the Cabibbo angle. The angles $\theta ^{\nu }_{ij} \cong (\theta _C)^n$ with $n > 2$ are set to zero. For example, in [54] the following sets of values of the angles in $U_e$ and $U_{\nu }$ have been used: $(\theta ^e_{12},\theta ^e_{13},\theta ^e_{23},\theta ^{\nu }_{12},\theta ^{\nu }_{13},\theta ^{\nu }_{23}) = (*,\pi /4,\pi /4,*,\pi /4,*)$ and $(*,*,\pi /4,\pi /4,*,*)$, where “$*$” means angles not exceeding $\theta _C$. None of these sets correspond to the cases studied by us. As a consequence, the sum rules for $\cos \delta$ derived in our work and in [54] are very different. In [54] the authors have also considered specific textures of the neutrino Majorana mass matrix leading to the two sets of values of the angles in $U_e$ and $U_{\nu }$ quoted above. However, these textures lead to values of $\sin ^2\theta _{23}$ or of $\sin ^2\theta _{12}$ which are strongly disfavoured by the current data. Although in [52, 53] a large variety of forms of $U_e$ and $U_{\nu }$ have been investigated, none of them corresponds to the forms studied by us, as can be inferred from the results on the values of the PMNS angles $\theta _{12}$, $\theta _{13}$ and $\theta _{23}$ obtained in [52, 53] and summarised in Table 2 in each of the two articles we have cited in [52, 53].

Our article is organised as follows. In Sect. 2 we consider the models which contain one rotation from the charged lepton sector, i.e., $\tilde{U}_e = R^{-1}_{12}(\theta ^e_{12})$, or $\tilde{U}_e = R^{-1}_{13}(\theta ^e_{13})$, and two rotations from the neutrino sector: $\tilde{U}_\nu = R_{23}(\theta ^{\nu }_{23}) \, R_{12}(\theta ^{\nu }_{12})$. In these cases the PMNS matrix reads

16 $$\begin{aligned} U = R_{ij}(\theta ^e_{ij}) \, \Psi \, R_{23}(\theta ^{\nu }_{23}) \, R_{12}(\theta ^{\nu }_{12}) \, Q_0 , \end{aligned}$$

with $(ij) = (12)$, (13). The matrix $\tilde{U}_{\nu }$ is assumed to have the following symmetry forms: TBM, BM (LC), GRA, GRB and HG. As we have already noted, for all these forms $\theta ^{\nu }_{23} = -\pi /4$, but we discuss also the general case of an arbitrary fixed value of $\theta ^{\nu }_{23}$. The forms listed above differ by the value of the angle $\theta ^{\nu }_{12}$, which for each of the forms of interest was given earlier. In Sect. 3 we analyse the models which contain two rotations from the charged lepton sector, i.e., $\tilde{U}_e = R^{-1}_{23}(\theta ^e_{23}) \, R^{-1}_{13}(\theta ^e_{13})$, or $\tilde{U}_e = R^{-1}_{13}(\theta ^e_{13}) \, R^{-1}_{12}(\theta ^e_{12})$, and^6^ two rotations from the neutrino sector, i.e.,

17 $$\begin{aligned}&U = R_{ij}(\theta ^e_{ij}) \, R_{kl}(\theta ^e_{kl}) \, \Psi \, R_{23}(\theta ^{\nu }_{23}) \, R_{12}(\theta ^{\nu }_{12}) \, Q_0 , \end{aligned}$$

with (*ij*)–$(kl) = (13)$–(23), (12)–(13). First we assume the angle $\theta ^{\nu }_{23}$ to correspond to the TBM, BM (LC), GRA, GRB and HG symmetry forms of $\tilde{U}_{\nu }$. After that we give the formulae for an arbitrary fixed value of this angle. Further, in Sect. 4, we generalise the schemes considered in Sect. 2 by allowing also a third rotation matrix to be present in $\tilde{U}_{\nu }$:

18 $$\begin{aligned} U = R_{ij}(\theta ^e_{ij}) \, \Psi \, R_{23}(\theta ^{\nu }_{23})\, R_{13}(\theta ^{\nu }_{13}) \, R_{12}(\theta ^{\nu }_{12}) \, Q_0 , \end{aligned}$$

with $$(ij) = (12)$$, (13), (23).

Using the sum rules for $\cos \delta$ derived in Sects. 2–4, in Sect. 5 we obtain predictions for $\cos \delta$, $\delta$ and $J_\mathrm{CP}$ for each of the models considered in the preceding sections. Section 6 contains summary of the results of the present study and conclusions.

We note finally that the titles of Sects. 2–4 and of their subsections reflect the rotations contained in the corresponding parametrisation, Eqs. (16)–(18).

## The cases of $\theta ^e_{ij}-(\theta ^\nu _{23}, \theta ^\nu _{12})$ rotations

In this section we derive the sum rules for $\cos \delta$ of interest in the case when the matrix $\tilde{U}_\nu = R_{23}(\theta ^{\nu }_{23})\, R_{12}(\theta ^{\nu }_{12})$ with fixed (e.g., symmetry) values of the angles $\theta ^{\nu }_{23}$ and $\theta ^{\nu }_{12}$, gets correction only due to one rotation from the charged lepton sector. The neutrino mixing matrix *U* has the form given in Eq. (16). We do not consider the cases of Eq. (16) (i) with $(ij) = (23)$, because the reactor angle $\theta _{13}$ does not get corrected and remains zero, and (ii) with $(ij) = (12)$ and $\theta ^{\nu }_{23} = -\pi /4$, which has been already analysed in detail in [45, 49].

### The scheme with $\theta ^e_{12}-(\theta ^\nu _{23}, \theta ^\nu _{12})$ rotations

For $\theta ^{\nu }_{23} = -\pi /4$ the sum rule for $\cos \delta$ in this case was derived in Ref. [45] and is given in Eq. (50) therein. Here we consider the case of an arbitrary fixed value of the angle $\theta ^{\nu }_{23}$. Using Eq. (16) with $(ij) = (12)$, one finds the following expressions for the mixing angles $\theta _{13}$ and $\theta _{23}$ of the standard parametrisation of the PMNS matrix:

19 $$\begin{aligned} \sin ^2 \theta _{13}&= |U_{e3}|^2 = \sin ^2 \theta ^e_{12} \sin ^2 \theta ^{\nu }_{23} ,\end{aligned}$$

20 $$\begin{aligned} \sin ^2 \theta _{23}&= \frac{|U_{\mu 3}|^2}{1-|U_{e3}|^2} = \frac{\sin ^2 \theta ^{\nu }_{23}-\sin ^2 \theta _{13}}{1 - \sin ^2 \theta _{13}} . \end{aligned}$$

Although Eq. (13) was derived in [45] for $\theta ^{\nu }_{23} = -\pi /4$ and $\tilde{U}_e = R^{-1}_{23}(\theta ^{e}_{23})R^{-1}_{12}(\theta ^{e}_{12})$, it is not difficult to convince oneself that it holds also in the case under discussion for an arbitrary fixed value of $\theta ^{\nu }_{23}$. The sum rule for $\cos \delta$ of interest, expressed in terms of the angles $\theta _{12}$, $\theta _{13}$, $\theta ^{\nu }_{12}$ and $\theta ^{\nu }_{23}$, can be obtained from Eq. (13) by using the expression for $\sin ^2 \theta _{23}$ given in Eq. (20). The result reads

21 $$\begin{aligned} \cos \delta&= \frac{(\cos 2 \theta _{13} - \cos 2 \theta ^{\nu }_{23})^{\frac{1}{2}}}{\sqrt{2}\sin 2\theta _{12}\sin \theta _{13} |\cos \theta ^{\nu }_{23}|}\, \left[ \cos 2\theta ^{\nu }_{12}+ \left( \sin ^2\theta _{12} \right. \right. \nonumber \\&\quad \left. \left. - \cos ^2\theta ^{\nu }_{12} \right) \!\times \! \frac{2\sin ^2 \theta ^{\nu }_{23}-(3+\cos 2 \theta ^{\nu }_{23}) \sin ^2\theta _{13}}{\cos 2 \theta _{13} - \cos 2 \theta ^{\nu }_{23}} \right] . \end{aligned}$$

Setting $\theta ^{\nu }_{23} = -\pi /4$ in Eq. (21), one reproduces the sum rule given in Eq. (50) in Ref. [45].

### The scheme with $\theta ^e_{13}-(\theta ^\nu _{23}, \theta ^\nu _{12})$ rotations 

In the present subsection we consider the parametrisation of the neutrino mixing matrix given in Eq. (16) with $(ij) = (13)$. In this set-up the phase $\psi$ in the matrix $\Psi$ is unphysical (it can be absorbed in the $\mu ^\pm$ field) and therefore effectively $\Psi = {{\mathrm{diag}}}(1, 1, e^{-i \omega })$. Using Eq. (16) with $(ij) = (13)$ and $\theta ^{\nu }_{23} = -\pi /4$ and the standard parametrisation of *U*, we get

22 $$\begin{aligned}&\sin ^2 \theta _{13} = |U_{e3}|^2 = \frac{1}{2} \sin ^2 \theta ^e_{13} ,\end{aligned}$$

23 $$\begin{aligned}&\sin ^2 \theta _{23} = \frac{|U_{\mu 3}|^2}{1-|U_{e3}|^2} = \frac{1}{2\,(1 - \sin ^2 \theta _{13})} , \end{aligned}$$

24 $$\begin{aligned}&\sin ^2 \theta _{12} = \frac{|U_{e2}|^2}{1-|U_{e3}|^2} = \frac{1}{1-\sin ^2 \theta _{13}} \Bigg [ \dfrac{1}{2} \sin ^2 \theta ^e_{13} \cos ^2 \theta ^{\nu }_{12} \nonumber \\&\quad + \cos ^2 \theta ^e_{13} \sin ^2 \theta ^{\nu }_{12}+ \frac{1}{\sqrt{2}} \sin 2 \theta ^e_{13} \cos \omega \sin \theta ^{\nu }_{12} \cos \theta ^{\nu }_{12} \Bigg ]. \end{aligned}$$

From Eqs. (22) and (24) we obtain an expression for $\cos \omega$ in terms of the measured mixing angles $\theta _{12}$, $\theta _{13}$ and the known $\theta ^{\nu }_{12}$:

25 $$\begin{aligned} \cos \omega= & {} \frac{1-\sin ^2 \theta _{13}}{\sin 2\theta ^{\nu }_{12} \sin \theta _{13} (1-2\sin ^2 \theta _{13})^{\frac{1}{2}}}\nonumber \\&\times \left[ \sin ^2 \theta _{12} - \sin ^2 \theta ^{\nu }_{12} - \cos 2\theta ^{\nu }_{12} \frac{\sin ^2 \theta _{13}}{1-\sin ^2 \theta _{13}} \right] .\nonumber \\ \end{aligned}$$

Further, one can find^7^ a relation between $\sin \delta$ ($\cos \delta$) and $\sin \omega$ ($\cos \omega$) by comparing the imaginary (the real) part of the quantity $U^*_{e1} U^*_{\mu 3} U_{e3} U_{\mu 1}$, written by using Eq. (16) with $(ij) = (13)$ and in the standard parametrisation of *U*. For the relation between $\sin \delta$ and $\sin \omega$ we get

26 $$\begin{aligned} \sin \delta = -\frac{\sin 2 \theta ^{\nu }_{12}}{\sin 2 \theta _{12}} \sin \omega . \end{aligned}$$

The sum rule for $\cos \delta$ of interest can be derived by substituting $\cos \omega$ from Eq. (25) in the relation between $\cos \delta$ and $\cos \omega$ (which is not difficult to derive and we do not give). We obtain

27 $$\begin{aligned} \cos \delta= & {} -\frac{(1-2\sin ^2 \theta _{13})^{\frac{1}{2}}}{\sin 2 \theta _{12} \sin \theta _{13}}\left[ \cos 2 \theta ^{\nu }_{12}+(\sin ^2 \theta _{12}\right. \nonumber \\&\left. -\cos ^2 \theta ^{\nu }_{12}) \frac{1-3\sin ^2 \theta _{13}}{1-2\sin ^2 \theta _{13}} \right] . \end{aligned}$$

We note that the expression for $\cos \delta$ thus found differs only by an overall minus sign from the analogous expression for $\cos \delta$ derived in [45] in the case of $(ij) = (12)$ rotation in the charged lepton sector (see Eq. (50) in [45]).

In Eq. (15) we have given the expression for the rephasing invariant $J_\mathrm{CP}$ in the standard parametrisation of the PMNS matrix. Below and in the next sections we give for completeness also the expressions of the $J_\mathrm{CP}$ factor in terms of the independent parameters of the set-up considered. In terms of the parameters $\omega$, $\theta ^e_{13}$ and $\theta ^{\nu }_{12}$ of the set-up discussed in the present subsection, $J_\mathrm{CP}$ is given by

28 $$\begin{aligned} J_\mathrm{CP} = -\frac{1}{8 \sqrt{2}} \sin \omega \sin 2\theta ^e_{13} \sin 2\theta ^{\nu }_{12} . \end{aligned}$$

In the case of an arbitrary fixed value of the angle $\theta ^{\nu }_{23}$ the expressions for the mixing angles $\theta _{13}$ and $\theta _{23}$ take the form

29 $$\begin{aligned} \sin ^2 \theta _{13}&= |U_{e3}|^2 = \sin ^2 \theta ^e_{13} \cos ^2 \theta ^{\nu }_{23} ,\end{aligned}$$

30 $$\begin{aligned} \sin ^2 \theta _{23}&= \frac{|U_{\mu 3}|^2}{1-|U_{e3}|^2} = \frac{\sin ^2 \theta ^{\nu }_{23}}{1 - \sin ^2 \theta _{13}} . \end{aligned}$$

The sum rule for $\cos \delta$ in this case can be obtained with a simpler procedure, namely, by using the expressions for the absolute value of the element $U_{\mu 1}$ of the PMNS matrix in the two parametrisations employed in the present subsection:

31 $$\begin{aligned} |U_{\mu 1}|&= |\cos \theta _{23} \sin \theta _{12} + e^{i \delta } \cos \theta _{12} \sin \theta _{13} \sin \theta _{23}|\nonumber \\&= |\cos \theta ^{\nu }_{23} \sin \theta ^{\nu }_{12}|. \end{aligned}$$

From Eq. (31) we get

32 $$\begin{aligned} \cos \delta&= -\frac{(\cos 2 \theta _{13} + \cos 2 \theta ^{\nu }_{23})^{\frac{1}{2}}}{\sqrt{2}\sin 2\theta _{12}\sin \theta _{13}|\sin \theta ^{\nu }_{23}|}\, \Bigg [ \cos 2\theta ^{\nu }_{12} \nonumber \\&\quad + \left( \sin ^2\theta _{12} - \cos ^2\theta ^{\nu }_{12} \right) \nonumber \\&\quad \times \frac{2\cos ^2 \theta ^{\nu }_{23}-(3-\cos 2 \theta ^{\nu }_{23}) \sin ^2\theta _{13}}{\cos 2 \theta _{13} + \cos 2 \theta ^{\nu }_{23}} \Bigg ]. \end{aligned}$$

We will use the sum rules for $\cos \delta$ derived in the present and the next two sections to obtain predictions for $\cos \delta$, $\delta$ and for the $J_\mathrm{CP}$ factor in Sect. 5.

## The cases of $(\theta ^e_{ij},\theta ^e_{kl})-(\theta ^\nu _{23}, \theta ^\nu _{12})$ rotations

As we have seen in the preceding section, in the case of one rotation from the charged lepton sector and for $\theta ^{\nu }_{23} = -\pi /4$, the mixing angle $\theta _{23}$ cannot deviate significantly from $\pi /4$ due to the smallness of the angle $\theta _{13}$. If the matrix $\tilde{U}_{\nu }$ has one of the symmetry forms considered in this study, the matrix $\tilde{U}_e$ has to contain at least two rotations in order to be possible to reproduce the current best fit values of the neutrino mixing parameters, quoted in Eqs. (3)–(5). This conclusion will remain valid if higher precision measurements of $\sin ^2\theta _{23}$ confirm that $\theta _{23}$ deviates significantly from $\pi /4$. In what follows we investigate different combinations of two rotations from the charged lepton sector and derive a sum rule for $\cos \delta$ in each set-up. We will not consider the case $(\theta ^e_{12},\theta ^e_{23})$–$(\theta ^\nu _{23}, \theta ^\nu _{12})$, because it has been thoroughly analysed in Refs. [45, 46, 49, 50], and, as we have already noted, the resulting sum rule Eq. (13) derived in [45] holds for an arbitrary fixed value of $\theta ^{\nu }_{23}$.

### The scheme with $(\theta ^e_{13},\theta ^e_{23})-(\theta ^\nu _{23}, \theta ^\nu _{12})$ rotations

Following the method used in Ref. [46], the PMNS matrix *U* from Eq. (17) with (*ij*) –$(kl) = (13)$– (23) can be cast in the form

33 $$\begin{aligned} U = R_{13}(\theta ^e_{13}) \, P_1 \, R_{23}(\hat{\theta }_{23}) \, R_{12}(\theta ^{\nu }_{12}) \, \hat{Q} , \end{aligned}$$

where the angle $\hat{\theta }_{23}$ is determined (i) for $\theta ^{\nu }_{23} = -\pi /4$ by

34 $$\begin{aligned} \sin ^2 \hat{\theta }_{23} = \frac{1}{2}(1 - \sin 2 \theta ^e_{23} \cos ( \omega - \psi )) , \end{aligned}$$

and (ii) for an arbitrary fixed value of $\theta ^{\nu }_{23}$ by

35 $$\begin{aligned} \sin ^2 \hat{\theta }_{23}= & {} \sin ^2 \theta ^e_{23} \cos ^2 \theta ^{\nu }_{23} + \cos ^2 \theta ^e_{23} \sin ^2 \theta ^{\nu }_{23}\nonumber \\&+\frac{1}{2} \sin 2 \theta ^e_{23} \sin 2 \theta ^{\nu }_{23} \cos ( \omega - \psi ) . \end{aligned}$$

The phase matrices $P_1$ and $\hat{Q}$ have the form

36 $$\begin{aligned} P_1= & {} {{\mathrm{diag}}}(1, 1, e^{-i \alpha }),\quad \text {and}\quad \hat{Q} = Q_1 \, Q_0,\nonumber \\&\text {with}~~Q_1 = {{\mathrm{diag}}}(1, 1, e^{i \beta }), \end{aligned}$$

where the phases $\alpha$ and $\beta$ are given by

37 $$\begin{aligned} \alpha= & {} \gamma + \psi + \omega ,~~\text {with}~~\gamma = \arg (e^{ -i \psi } \cos \theta ^e_{23} \sin \theta ^{\nu }_{23}\nonumber \\&+ e^{-i \omega } \sin \theta ^e_{23} \cos \theta ^{\nu }_{23}), \end{aligned}$$

38 $$\begin{aligned} \beta= & {} \gamma - \phi ,~~\text {where}~~\phi = \arg (e^{ -i \psi } \cos \theta ^e_{23} \cos \theta ^{\nu }_{23}\nonumber \\&- e^{-i \omega } \sin \theta ^e_{23} \sin \theta ^{\nu }_{23}). \end{aligned}$$

Using Eq. (33) and the standard parametrisation of *U*, we find

39 $$\begin{aligned} \sin ^2 \theta _{13}&= |U_{e3}|^2 = \sin ^2 \theta ^e_{13} \cos ^2 \hat{\theta }_{23} ,\end{aligned}$$

40 $$\begin{aligned} \sin ^2 \theta _{23}&= \frac{|U_{\mu 3}|^2}{1-|U_{e3}|^2} = \dfrac{\sin ^2 \hat{\theta }_{23}}{1 - \sin ^2 \theta _{13}} ,\end{aligned}$$

41 $$\begin{aligned} \sin ^2 \theta _{12}&= \frac{|U_{e2}|^2}{1-|U_{e3}|^2} = \dfrac{1}{1 - \sin ^2 \theta _{13}} \bigg [ \cos ^2 \theta ^e_{13} \sin ^2 \theta ^{\nu }_{12} \nonumber \\&\quad - \dfrac{1}{2} \sin \hat{\theta }_{23} \sin 2 \theta ^e_{13} \sin 2 \theta ^{\nu }_{12} \cos \alpha \nonumber \\&\quad + \cos ^2 \theta ^{\nu }_{12} \sin ^2 \theta ^e_{13} \sin ^2 \hat{\theta }_{23} \bigg ] . \end{aligned}$$

The first two equations allow one to express $\theta ^e_{13}$ and $\hat{\theta }_{23}$ in terms of $\theta _{13}$ and $\theta _{23}$. Equation (41) allows us to find $\cos \alpha$ as a function of the PMNS mixing angles $\theta _{12}$, $\theta _{13}$, $\theta _{23}$ and the angle $\theta ^{\nu }_{12}$:

42 $$\begin{aligned} \cos \alpha = 2 \, \frac{\sin ^2 \theta ^{\nu }_{12} \cos ^2 \theta _{23} + \cos ^2 \theta ^{\nu }_{12} \sin ^2 \theta _{23} \sin ^2 \theta _{13} - \sin ^2 \theta _{12} \left( 1 - \sin ^2 \theta _{23} \cos ^2 \theta _{13}\right) }{\sin 2 \theta ^{\nu }_{12} \sin 2 \theta _{23} \sin \theta _{13}} . \end{aligned}$$

The relation^8^ between $\sin \delta$ ($\cos \delta$) and $\sin \alpha$ ($\cos \alpha$) can be found by comparing the imaginary (the real) part of the quantity $U^*_{e1} U^*_{\mu 3} U_{e3} U_{\mu 1}$, written using Eq. (33) and using the standard parametrisation of *U*:

43 $$\begin{aligned} \sin \delta&= \dfrac{\sin 2 \theta ^{\nu }_{12}}{\sin 2 \theta _{12}} \sin \alpha , \end{aligned}$$

44 $$\begin{aligned} \cos \delta&= \dfrac{\sin 2 \theta ^{\nu }_{12}}{\sin 2 \theta _{12}} \cos \alpha \nonumber \\&\quad - \dfrac{\sin \theta _{13}}{\sin 2 \theta _{12}} \tan \theta _{23} (\cos 2 \theta _{12} + \cos 2 \theta ^{\nu }_{12}). \end{aligned}$$

The sum rule expression for $\cos \delta$ as a function of the mixing angles $\theta _{12}$, $\theta _{13}$, $\theta _{23}$ and $\theta ^{\nu }_{12}$, with $\theta ^{\nu }_{12}$ having an arbitrary fixed value, reads

45 $$\begin{aligned} \cos \delta&= -\frac{\cot \theta _{23}}{\sin 2\theta _{12}\sin \theta _{13}}\, [\cos 2\theta ^{\nu }_{12}+ (\sin ^2\theta _{12} - \cos ^2\theta ^{\nu }_{12} )\nonumber \\&\quad \times (1 - \tan ^2\theta _{23}\,\sin ^2\theta _{13} ) ]. \end{aligned}$$

This sum rule for $\cos \delta$ can be obtained formally from the r.h.s. of Eq. (13) by interchanging $\tan \theta _{23}$ and $\cot \theta _{23}$ and by multiplying it by $(-1)$. Thus, in the case of $\theta _{23} = \pi /4$, the predictions for $\cos \delta$ in the case under consideration will differ from those obtained using Eq. (13) only by a sign. We would like to emphasise that, as the sum rule in Eq. (13), the sum rule in Eq. (45) is valid for any fixed value of $\theta ^{\nu }_{23}$.

The $J_\mathrm{CP}$ factor has the following form in the parametrisation of the PMNS matrix employed in the present subsection:

46 $$\begin{aligned} J_\mathrm{CP} = \frac{1}{8} \sin 2 \theta ^e_{13} \sin 2 \theta ^{\nu }_{12} \sin 2 \hat{\theta }_{23} \cos \hat{\theta }_{23} \sin \alpha . \end{aligned}$$

### The scheme with $(\theta ^e_{12},\theta ^e_{13}) \,$–$\, (\theta ^\nu _{23}, \theta ^\nu _{12})$ rotations

In this subsection we consider the parametrisation of the matrix *U* defined in Eq. (17) with (*ij*) –$(kl) = (12)$– (13) under the assumption of vanishing $\omega$, i.e., $\Psi = {{\mathrm{diag}}}(1, e^{-i \psi },1)$. In the case of non-fixed $\omega$ it is impossible to express $\cos \delta$*only* in terms of the independent angles of the scheme. We will comment more on this case later.

Using the parametrisation given in Eq. (17) with $\theta ^{\nu }_{23} = -\pi /4$ and $\omega = 0$ and the standard one, we find

47 $$\begin{aligned} \sin ^2 \theta _{13}&= |U_{e3}|^2 = \dfrac{1}{2} \sin ^2 \theta ^e_{12} + \dfrac{1}{2} \cos ^2 \theta ^e_{12} \sin ^2 \theta ^e_{13} - X_{\psi }, \end{aligned}$$

48 $$\begin{aligned} \sin ^2 \theta _{23}&= \frac{|U_{\mu 3}|^2}{1-|U_{e3}|^2} = \dfrac{1}{\cos ^2 \theta _{13}}\nonumber \\&\quad \times \bigg [ \dfrac{1}{2} \cos ^2 \theta ^e_{12} + \dfrac{1}{2} \sin ^2 \theta ^e_{12} \sin ^2 \theta ^e_{13} + X_{\psi } \bigg ], \end{aligned}$$

49 $$\begin{aligned} \sin ^2 \theta _{12}&= \frac{|U_{e2}|^2}{1-|U_{e3}|^2} = \dfrac{\zeta \sin ^2 \theta ^e_{12} + \xi }{1-\sin ^2 \theta _{13}}, \end{aligned}$$

where

50 $$\begin{aligned}&X_{\psi } = \dfrac{1}{2} \sin 2 \theta ^e_{12} \sin \theta ^e_{13} \cos \psi , \end{aligned}$$

51 $$\begin{aligned}&\zeta = \cos ^2 \theta ^e_{13} \cos 2 \theta ^{\nu }_{12} + \dfrac{1}{4\sqrt{2}} \sin 2\theta ^{\nu }_{12} \cot \theta ^e_{13} (3 \cos 2 \theta ^e_{13} \!-\! 1), \end{aligned}$$

52 $$\begin{aligned}&\xi = \cos ^2 \theta ^e_{13} \sin ^2 \theta ^{\nu }_{12} + \dfrac{1}{2} (\cos 2 \theta _{13} - \cos 2 \theta ^e_{13}) \cos ^2 \theta ^{\nu }_{12} \nonumber \\&\qquad + \dfrac{1}{2 \sqrt{2}} \sin 2 \theta ^{\nu }_{12} (3 \cos \theta ^e_{13} \sin \theta ^e_{13} -2 \cot \theta ^e_{13} \sin ^2 \theta _{13}). \end{aligned}$$

The dependence on $\cos \psi$ in Eq. (49) has been eliminated by solving Eq. (47) for $X_{\psi }$. It follows from Eqs. (47) and (48) that $\sin ^2\theta ^e_{13}$ is a function of the known mixing angles $\theta _{13}$ and $\theta _{23}$:

53 $$\begin{aligned} \sin ^2 \theta ^e_{13} = 1 - 2 \cos ^2 \theta _{13} \cos ^2 \theta _{23}\, . \end{aligned}$$

Inverting the formula for $\sin ^2 \theta _{12}$ allows us to find $\sin ^2 \theta ^e_{12}$, which is given by

54 $$\begin{aligned} \sin ^2 \theta ^e_{12}&= [ 4 [\cos 2 \theta ^{\nu }_{12} (\cos 2 \theta ^e_{13} + \sin ^2 \theta _{13})\nonumber \\&\quad - \cos 2 \theta _{12} \cos ^2 \theta _{13} ] \tan \theta ^e_{13} + \sqrt{2} \sin 2 \theta ^{\nu }_{12} \nonumber \\&\quad \times (3 \cos 2 \theta ^e_{13} \!-\! 2 \cos 2 \theta _{13} \!-\! 1) ] [4 \cos 2 \theta ^{\nu }_{12} \sin 2 \theta ^e_{13}\nonumber \\&\quad + \sqrt{2}(3 \cos 2 \theta ^e_{13} - 1) \sin 2 \theta ^{\nu }_{12} ]^{-1} . \end{aligned}$$

Using Eqs. (47) and  (54) we can write $\cos \psi$ in terms of the standard parametrisation mixing angles and the known $\theta ^e_{13}$ and $\theta ^{\nu }_{12}$:

55 $$\begin{aligned} \cos \psi = \frac{\sin ^2 \theta ^e_{12} + \cos ^2 \theta ^e_{12} \sin ^2 \theta ^e_{13} -2 \sin ^2 \theta _{13}}{\sin 2 \theta ^e_{12} \sin \theta ^e_{13}}. \end{aligned}$$

We find the relation between $\sin \delta$ and $\sin \psi$ by employing again the standard procedure of comparing the expressions of the $J_\mathrm{CP}$ factor, $J_\mathrm{CP} = \mathrm{Im}(U^*_{e1} U^*_{\mu 3} U_{e3} U_{\mu 1})$, in the two parametrisations—the standard one and that defined in Eq. (17) (with $\theta ^{\nu }_{23} = -\pi /4$ and $\omega = 0$):

56 $$\begin{aligned} \sin \delta= & {} \frac{\sin 2 \theta ^e_{12} \sin \psi }{4 \sin 2 \theta _{12} \sin 2 \theta _{13} \sin \theta _{23}}\nonumber \\&\times [ 2 \sqrt{2} \sin 2 \theta ^e_{13} \cos 2 \theta ^{\nu }_{12} \!+\! (3 \cos 2 \theta ^e_{13} \!-\! 1) \sin 2 \theta ^{\nu }_{12}],\nonumber \\ \end{aligned}$$

where $\sin 2\theta ^e_{12}$ ($\sin 2\theta ^e_{13}$ and $\cos 2 \theta ^e_{13}$) can be expressed in terms of $\theta _{12}$, $\theta _{13}$, $\theta _{23}$ and $\theta ^{\nu }_{12}$ ($\theta _{13}$ and $\theta _{23}$) using Eq. (54) (Eq. (53)).

We use a much simpler procedure to find $\cos \delta$. Namely, we compare the expressions for the absolute value of the element $U_{\tau 1}$ of the PMNS matrix in the standard parametrisation and in the symmetry related one, Eq. (17) with $\theta ^{\nu }_{23} = -\pi /4$ and $\omega = 0$, considered in the present subsection:

57 $$\begin{aligned} |U_{\tau 1}|&= |\sin \theta _{23} \sin \theta _{12} - \sin \theta _{13} \cos \theta _{23} \cos \theta _{12} e^{i \delta }|\nonumber \\&=|\sin \theta ^e_{13} \cos \theta ^{\nu }_{12} + \frac{1}{\sqrt{2}} \cos \theta ^e_{13} \sin \theta ^{\nu }_{12}| . \end{aligned}$$

From the above equation we get for $\cos \delta$:

58 $$\begin{aligned} \cos \delta&= -\frac{2}{\sin 2 \theta _{12} \sin 2 \theta _{23} \sin \theta _{13}}\nonumber \\&\quad \times \bigg [ \cos ^2 \theta _{23} \sin ^2 \theta _{12} \sin ^2 \theta _{13} + \cos ^2 \theta _{12} \sin ^2 \theta _{23} \nonumber \\&\quad - \bigg ( \sqrt{\cos ^2 \theta _{13} \cos ^2 \theta _{23}} \cos \theta ^{\nu }_{12}\nonumber \\ {}&\quad - \kappa \sqrt{1- 2 \cos ^2 \theta _{13} \cos ^2 \theta _{23}} \sin \theta ^{\nu }_{12} \bigg )^2 \, \bigg ] , \end{aligned}$$

where $\kappa = 1$ if $\theta ^e_{13}$ belongs to the first or third quadrant, and $\kappa = -1$ if $\theta ^e_{13}$ is in the second or the fourth one. In the parametrisation under discussion, Eq. (17) with (*ij*) –$(kl) = (12)$– (13), $\theta ^{\nu }_{23} = -\pi /4$ and $\omega = 0$, we have

59 $$\begin{aligned} J_\mathrm{CP}= & {} \frac{\sqrt{2}}{32} \cos \theta ^e_{13} \sin 2 \theta ^e_{12} ( 2 \sqrt{2} \cos 2 \theta ^{\nu }_{12} \sin 2 \theta ^e_{13}\nonumber \\&+ (3 \cos 2 \theta ^e_{13} - 1) \sin 2 \theta ^{\nu }_{12} ) \sin \psi . \end{aligned}$$

In the case of non-vanishing $\omega$, using the same method and Eq. (53), which also holds for $\omega \ne 0$, allows us to show that $\cos \delta$ is a function of $\cos \omega$ as well:

60 $$\begin{aligned} \cos \delta&= -\frac{2 \cos ^2 \theta _{23} }{\sin 2 \theta _{12} \sin 2 \theta _{23} \sin \theta _{13}}\nonumber \\&\quad \times \!\bigg [\! (1 \!-\! 2 \cos ^2 \theta _{13} \cos ^2 \theta _{23}) \frac{\cos ^2 \theta ^{\nu }_{12}}{\cos ^2 \theta _{23} } \!-\! \sin ^2 \theta _{12} \tan ^2 \theta _{23} \nonumber \\&\quad + ( \cos ^2 \theta _{13} \sin ^2 \theta ^{\nu }_{12} - \cos ^2 \theta _{12} \sin ^2 \theta _{13})\nonumber \\&\quad + \kappa \frac{\cos \theta _{13}}{\cos \theta _{23}} \sqrt{1 - 2 \cos ^2 \theta _{13} \cos ^2 \theta _{23}} \cos \omega \sin 2 \theta ^{\nu }_{12} \bigg ] . \end{aligned}$$

Finally, we generalise Eq. (60) to the case of an arbitrary fixed value of $\theta ^{\nu }_{23}$. In this case

61 $$\begin{aligned} \sin ^2 \theta ^e_{13} = \frac{1 - \cos ^2 \theta _{13} \cos ^2 \theta _{23} - \sin ^2 \theta ^{\nu }_{23}}{\cos ^2 \theta ^{\nu }_{23}} , \end{aligned}$$

and Eqs. (57) and (60) read

62 $$\begin{aligned} |U_{\tau 1}|&= |\sin \theta _{23} \sin \theta _{12} - \sin \theta _{13} \cos \theta _{23} \cos \theta _{12} e^{i \delta }|\nonumber \\&=|\cos \theta ^{\nu }_{12} \sin \theta ^e_{13} - e^{-i \omega }\cos \theta ^e_{13} \sin \theta ^{\nu }_{12} \sin \theta ^{\nu }_{23}| , \end{aligned}$$

63 $$\begin{aligned} \cos \delta&= \dfrac{1}{\sin 2 \theta _{12} \sin 2 \theta _{23} \sin \theta _{13}}\nonumber \\ {}&\quad \times \bigg [ \frac{2 \kappa \cos \omega \sin 2 \theta ^{\nu }_{12} \sin \theta ^{\nu }_{23} \cos \theta _{13} \cos \theta _{23}}{\cos ^2 \theta ^{\nu }_{23}}\nonumber \\&\quad \times (\cos ^2 \theta ^{\nu }_{23} - \cos ^2 \theta _{13} \cos ^2 \theta _{23})^{\frac{1}{2}} \nonumber \\&\quad - \cos 2 \theta ^{\nu }_{12} \Bigg (1 - \frac{\cos ^2 \theta _{13} \cos ^2 \theta _{23}}{\cos ^2 \theta ^{\nu }_{23} }(\sin ^2 \theta ^{\nu }_{23} + 1) \Bigg )\nonumber \\&\quad +\cos 2 \theta _{12} (\cos ^2 \theta _{23} \sin ^2 \theta _{13} - \sin ^2 \theta _{23} ) \bigg ] . \end{aligned}$$

It follows from the results for $\cos \delta$ obtained for $\cos \omega \ne 0$, Eqs. (60) and (63), that in the case analysed in the present subsection one can obtain predictions for $\cos \delta$ only in theoretical models in which the value of the phase $\omega$ is fixed by the model.

## The cases of $\theta ^e_{ij}-(\theta ^\nu _{23}, \theta ^\nu _{13}, \theta ^\nu _{12})$ rotations

We consider next a generalisation of the cases analysed in Sect. 2 in the presence of a third rotation matrix in $\tilde{U}_{\nu }$ arising from the neutrino sector, i.e., we employ the parametrisation of *U* given in Eq. (18). Non-zero values of $\theta ^{\nu }_{13}$ are inspired by certain types of flavour symmetries (see, e.g., [63–67]). In the case of $\theta ^{\nu }_{12} = \theta ^{\nu }_{23} = - \pi /4$ and $\theta ^{\nu }_{13} = \sin ^{-1} (1 / 3)$, for instance, we have the so-called tri-permuting (TP) pattern, which was proposed and studied in [63]. In the statistical analysis of the predictions for $\cos \delta$, $\delta$ and the $J_\mathrm{CP}$ factor we will perform in Sect. 5, we will consider three representative values of $\theta ^{\nu }_{13}$ discussed in the literature: $\theta ^{\nu }_{13} = \pi /20,~\pi /10$ and $\sin ^{-1} (1 / 3)$.

For the parametrisation of the matrix *U* given in Eq. (18) with $(ij) = (23)$, no constraints on the phase $\delta$ can be obtained. Indeed, after we recast *U* in the form

64 $$\begin{aligned} U = R_{23}(\hat{\theta }_{23}) \, Q_1 \, R_{13}(\theta ^{\nu }_{13})\, R_{12}(\theta ^{\nu }_{12}) \, Q_0 , \end{aligned}$$

where $\sin ^2 \hat{\theta }_{23}$ and $Q_1$ are given in Eqs. (35) and (36), respectively, we find employing a similar procedure used in the previous sections:

65 $$\begin{aligned} \sin ^2 \theta _{13}= & {} \sin ^2 \theta ^{\nu }_{13}, \quad \sin ^2 \theta _{23} = \sin ^2 \hat{\theta }_{23}, \nonumber \\ \sin ^2 \theta _{12}= & {} \sin ^2 \theta ^{\nu }_{12}, \quad \sin \delta = \sin \beta . \end{aligned}$$

Thus, there is no correlation between the Dirac CPV phase $\delta$ and the mixing angles in this set-up.

### The scheme with $\theta ^e_{12} -(\theta ^\nu _{23}, \theta ^\nu _{13}, \theta ^\nu _{12})$ rotations

In the parametrisation of the matrix *U* given in Eq. (18) with $(ij) = (12)$, the phase $\omega$ in the matrix $\Psi$ is unphysical [it “commutes” with $R_{12}(\theta ^e_{12})$ and can be absorbed by the $\mu ^\pm$ field]. Hence, the matrix $\Psi$ contains only one physical phase $\phi$, $\Psi = {{\mathrm{diag}}}\, (1, e ^{i \phi }, 1)$, and $\phi \equiv - \psi$. Taking this into account and using Eq. (18) with $(ij) = (12)$ and $\theta ^{\nu }_{23} = -\pi /4$, we get the following expressions for $\sin ^2\theta _{13}$, $\sin ^2\theta _{23}$ and $\sin ^2\theta _{12}$:

66 $$\begin{aligned} \sin ^2 \theta _{13}&= |U_{e3}|^2 = \frac{1}{2} \sin ^2 \theta ^e_{12} \cos ^2 \theta ^{\nu }_{13}\nonumber \\&\quad + \cos ^2 \theta ^e_{12} \sin ^2 \theta ^{\nu }_{13} - X_{12} \sin \theta ^{\nu }_{13} , \end{aligned}$$

67 $$\begin{aligned} \sin ^2 \theta _{23}&= \frac{|U_{\mu 3}|^2}{1-|U_{e3}|^2} = 1 - \frac{\cos ^2 \theta ^{\nu }_{13}}{2\,(1 - \sin ^2 \theta _{13})} , \end{aligned}$$

68 $$\begin{aligned} \sin ^2 \theta _{12}&= \frac{|U_{e2}|^2}{1-|U_{e3}|^2} = \frac{1}{1-\sin ^2 \theta _{13}}\nonumber \\&\quad \times \bigg [ \dfrac{1}{2} \sin ^2 \theta ^e_{12} ( \cos \theta ^{\nu }_{12} + \sin \theta ^{\nu }_{12} \sin \theta ^{\nu }_{13})^2 \nonumber \\&\quad + \cos ^2 \theta ^e_{12} \cos ^2 \theta ^{\nu }_{13} \sin ^2 \theta ^{\nu }_{12} + X_{12} \sin \theta ^{\nu }_{12} \nonumber \\&\quad \times ( \cos \theta ^{\nu }_{12} + \sin \theta ^{\nu }_{12} \sin \theta ^{\nu }_{13}) \bigg ] , \end{aligned}$$

where

69 $$\begin{aligned} X_{12} = \frac{1}{\sqrt{2}} \sin 2 \theta ^e_{12} \cos \theta ^{\nu }_{13} \cos \phi . \end{aligned}$$

Solving Eq. (66) for $X_{12}$ and inserting the solution in Eq. (68), we find $\sin ^2 \theta _{12}$ as a function of $\theta _{13}$, $\theta ^{\nu }_{12}$, $\theta ^{\nu }_{13}$ and $\theta ^e_{12}$:

70 $$\begin{aligned} \sin ^2 \theta _{12} = \frac{\alpha \sin ^2 \theta ^e_{12} + \beta }{1-\sin ^2 \theta _{13}} . \end{aligned}$$

Here the parameters $\alpha$ and $\beta$ are given by

71 $$\begin{aligned} \alpha&= \frac{1}{4} \left[ 2\cos 2 \theta ^{\nu }_{12} + \sin 2 \theta ^{\nu }_{12} \frac{\cos ^2 \theta ^{\nu }_{13} }{\sin \theta ^{\nu }_{13} } \right] ,\end{aligned}$$

72 $$\begin{aligned} \beta&= \sin \theta ^{\nu }_{12} \left[ \cos ^2 \theta _{13} \sin \theta ^{\nu }_{12} \!+\! \cos \theta ^{\nu }_{12} \left( \! \sin \theta ^{\nu }_{13} \!-\! \frac{\sin ^2 \theta _{13}}{\sin \theta ^{\nu }_{13}} \!\right) \!\right] . \end{aligned}$$

Inverting the formula for $\sin ^2 \theta _{12}$ allows us to express $\sin ^2 \theta ^e_{12}$ in terms of $\theta _{12}$, $\theta _{13}$, $\theta ^{\nu }_{12}$, $\theta ^{\nu }_{13}$:

73 $$\begin{aligned} \sin ^2 \theta ^e_{12}= \frac{2 \cos ^2 \theta _{13}\sin \theta ^{\nu }_{13} (\sin ^2 \theta _{12} - \sin ^2 \theta ^{\nu }_{12}) + \sin 2 \theta ^{\nu }_{12} \sin ^2 \theta _{13} - \sin 2\theta ^{\nu }_{12} \sin ^2 \theta ^{\nu }_{13}}{\cos 2 \theta ^{\nu }_{12} \sin \theta ^{\nu }_{13} + \cos \theta ^{\nu }_{12} \sin \theta ^{\nu }_{12} \cos ^2 \theta ^{\nu }_{13}} . \end{aligned}$$

In the limit of vanishing $\theta ^{\nu }_{13}$ we have $\sin ^2 \theta ^e_{12} = 2 \sin ^2 \theta _{13}$, which corresponds to the case of negligible $\theta ^e_{23}$ considered in [45].

Using Eq. (68), one can express $\cos \phi$ in terms of the “standard” mixing angles $\theta _{12}$, $\theta _{13}$ and the angles $\theta ^e_{12}$, $\theta ^{\nu }_{12}$ and $\theta ^{\nu }_{13}$ which are assumed to have known values:

74 $$\begin{aligned} \cos \phi&= [2 \cos ^2 \theta _{13} (\sin \theta ^e_{12})^{-2} (\sin \theta ^{\nu }_{12})^{-2} \sin ^2 \theta _{12}\nonumber \\&\quad - 2 \cos ^2 \theta ^{\nu }_{13} \cot ^2 \theta ^e_{12} - (\cot \theta ^{\nu }_{12} + \sin \theta ^{\nu }_{13})^2 ] \nonumber \\&\quad \times (\cos \theta ^{\nu }_{13})^{-1} \tan \theta ^e_{12} [ 2 \sqrt{2} (\cot \theta ^{\nu }_{12} + \sin \theta ^{\nu }_{13})]^{-1} . \end{aligned}$$

We note that from the requirements $(0 < \sin ^2 \theta ^e_{12} < 1) \wedge (-1 < \cos \phi < 1)$ one can obtain for a given $\theta ^{\nu }_{13}$, each of the symmetry values of $\theta ^{\nu }_{12}$ considered and $\theta ^{\nu }_{23} = -\pi /4$, lower and upper bounds on the value of $\sin ^2 \theta _{12}$. These bounds will be discussed in Sect. 5.2. Comparing the expressions for $J_\mathrm{CP} = \mathrm{Im}(U^*_{e1} U^*_{\mu 3} U_{e3} U_{\mu 1})$, obtained using Eq. (18) with $(ij) = (12)$ and $\theta ^{\nu }_{23} = -\pi /4$, and in the standard parametrisation of *U*, one gets the relation between $\sin \phi$ and $\sin \delta$:

75 $$\begin{aligned} \sin \delta&= -\frac{\sin 2 \theta ^e_{12}}{2 \sin 2 \theta _{12} \sin 2 \theta _{13} \sin \theta _{23}}\nonumber \\&\quad \times [ \cos ^2 \theta ^{\nu }_{13}\sin 2 \theta ^{\nu }_{12} + 2 \cos 2 \theta ^{\nu }_{12} \sin \theta ^{\nu }_{13} ] \sin \phi . \end{aligned}$$

Similarly to the method employed in the previous section, we use the equality of the expressions for $|U_{\tau 1}|$ in the two parametrisations in order to derive the sum rule for $\cos \delta$ of interest:

76 $$\begin{aligned} |U_{\tau 1}|&= |\sin \theta _{23} \sin \theta _{12} - \sin \theta _{13} \cos \theta _{23} \cos \theta _{12} e^{i \delta }|\nonumber \\&=\frac{1}{\sqrt{2}} |\sin \theta ^{\nu }_{12} + \cos \theta ^{\nu }_{12} \sin \theta ^{\nu }_{13}| . \end{aligned}$$

From the above equation we find the following sum rule for $\cos \delta$:

77 $$\begin{aligned} \cos \delta&= \frac{1}{\sin 2\theta _{12} \sin \theta _{13} |\cos \theta ^{\nu }_{13}| (1 - 2\sin ^2\theta _{13} + \sin ^2\theta ^{\nu }_{13})^{\frac{1}{2}}}\nonumber \\&\quad \times [(1 - 2\sin ^2\theta _{13} + \sin ^2\theta ^{\nu }_{13}) \sin ^2 \theta _{12} \nonumber \\&\quad + \cos ^2\theta _{12}\sin ^2\theta _{13}\cos ^2\theta ^{\nu }_{13}\nonumber \\&\quad - \cos ^2\theta _{13} (\sin \theta ^{\nu }_{12} + \cos \theta ^{\nu }_{12} \sin \theta ^{\nu }_{13})^2]. \end{aligned}$$

For $\theta ^{\nu }_{13} = 0$ this sum rule reduces to the sum rule for $\cos \delta$ given in Eq. (50) in [45].

In the parametrisation of the PMNS matrix considered in this subsection, the rephasing invariant $J_\mathrm{CP}$ has the form

78 $$\begin{aligned} J_\mathrm{CP}= & {} -\frac{1}{8 \sqrt{2}} \sin \phi \cos \theta ^{\nu }_{13} \sin 2\theta ^e_{12}\nonumber \\&\times [\cos ^2 \theta ^{\nu }_{13} \sin 2\theta ^{\nu }_{12} + 2 \sin \theta ^{\nu }_{13} \cos 2\theta ^{\nu }_{12}] . \end{aligned}$$

In the case when $\theta ^{\nu }_{23}$ has a fixed value which differs from $-\pi /4$, the expression for $\sin ^2 \theta _{23}$, Eq. (67), changes as follows:

79 $$\begin{aligned} \sin ^2 \theta _{23}&= \frac{|U_{\mu 3}|^2}{1-|U_{e3}|^2} = 1 - \frac{\cos ^2 \theta ^{\nu }_{23} \cos ^2 \theta ^{\nu }_{13}}{1 - \sin ^2 \theta _{13}} . \end{aligned}$$

Equations (76) and (77) are also modified:

80 $$\begin{aligned} |U_{\tau 1}|&= |\sin \theta _{23} \sin \theta _{12} - \sin \theta _{13} \cos \theta _{23} \cos \theta _{12} e^{i \delta }|\nonumber \\&= |\sin \theta ^{\nu }_{12} \sin \theta ^{\nu }_{23} - \cos \theta ^{\nu }_{23} \cos \theta ^{\nu }_{12} \sin \theta ^{\nu }_{13}| , \end{aligned}$$

and

81 $$\begin{aligned}&\cos \delta \nonumber \\&\quad = \frac{1}{\sin 2 \theta _{12} \sin \theta _{13} | \cos \theta ^{\nu }_{13} \cos \theta ^{\nu }_{23}| (\cos ^2 \theta _{13} \!-\! \cos ^2 \theta ^{\nu }_{13} \cos ^2 \theta ^{\nu }_{23})^{\frac{1}{2}}} \nonumber \\&\qquad \times [ (\cos ^2 \theta _{13} - \cos ^2 \theta ^{\nu }_{13} \cos ^2 \theta ^{\nu }_{23}) \sin ^2 \theta _{12} \nonumber \\&\qquad + \cos ^2 \theta _{12} \sin ^2 \theta _{13} \cos ^2 \theta ^{\nu }_{13} \cos ^2 \theta ^{\nu }_{23} \nonumber \\&\qquad - \cos ^2 \theta _{13} ( \cos \theta ^{\nu }_{12} \sin \theta ^{\nu }_{13} \cos \theta ^{\nu }_{23} - \sin \theta ^{\nu }_{12} \sin \theta ^{\nu }_{23})^2 ] . \end{aligned}$$

In the case of bi-trimaximal mixing [65, 66], i.e., for $\theta ^\nu _{12} = \theta ^{\nu }_{23} = \tan ^{-1} (\sqrt{3} - 1)$ and $\theta ^\nu _{13} = \sin ^{-1} ((3 - \sqrt{3})/6)$, the sum rule we have derived reduces to the sum rule obtained in [68]. However, this case is statistically disfavoured by the current global neutrino oscillation data.

### The scheme with $\theta ^e_{13}-(\theta ^\nu _{23}, \theta ^\nu _{13}, \theta ^\nu _{12})$ rotations

Here we switch to the parametrisation of the matrix *U* given in Eq. (18) with $(ij) = (13)$. Now the phase $\psi$ in the matrix $\Psi$ is unphysical, and $\Psi = {{\mathrm{diag}}}(1, 1, e^{-i \omega })$. Fixing $\theta ^{\nu }_{23} = -\pi /4$ and using also the standard parametrisation of *U*, we find

82 $$\begin{aligned} \sin ^2 \theta _{13}&= |U_{e3}|^2 = \frac{1}{2} \sin ^2 \theta ^e_{13} \cos ^2 \theta ^{\nu }_{13} + \cos ^2 \theta ^e_{13} \sin ^2 \theta ^{\nu }_{13}\nonumber \\&\quad + X_{13} \sin \theta ^{\nu }_{13} , \end{aligned}$$

83 $$\begin{aligned} \sin ^2 \theta _{23}&= \frac{|U_{\mu 3}|^2}{1-|U_{e3}|^2} = \frac{\cos ^2 \theta ^{\nu }_{13}}{2\,(1 - \sin ^2 \theta _{13})} , \end{aligned}$$

84 $$\begin{aligned} \sin ^2 \theta _{12}&= \frac{|U_{e2}|^2}{1-|U_{e3}|^2} \nonumber \\&= \frac{1}{1\!-\!\sin ^2 \theta _{13}} \bigg [ \dfrac{1}{2} \sin ^2 \theta ^e_{13} \left( \cos \theta ^{\nu }_{12} \!-\! \sin \theta ^{\nu }_{12} \sin \theta ^{\nu }_{13}\right) ^2 \nonumber \\&\quad + \cos ^2 \theta ^e_{13} \cos ^2 \theta ^{\nu }_{13} \sin ^2 \theta ^{\nu }_{12} + X_{13} \sin \theta ^{\nu }_{12} \nonumber \\&\quad \times ( \cos \theta ^{\nu }_{12} - \sin \theta ^{\nu }_{12} \sin \theta ^{\nu }_{13}) \bigg ] . \end{aligned}$$

Here

85 $$\begin{aligned} X_{13} = \frac{1}{\sqrt{2}} \sin 2 \theta ^e_{13} \cos \theta ^{\nu }_{13} \cos \omega . \end{aligned}$$

Solving Eq. (82) for $X_{13}$ and inserting the solution in Eq. (84), it is not dificult to find $\sin ^2 \theta _{12}$ as a function of $\theta _{13}$, $\theta ^{\nu }_{12}$, $\theta ^{\nu }_{13}$ and $\theta ^e_{13}$:

86 $$\begin{aligned} \sin ^2 \theta _{12} = \frac{\rho \sin ^2 \theta ^e_{13} + \eta }{1-\sin ^2 \theta _{13}} , \end{aligned}$$

where $\rho$ and $\eta$ are given by

87 $$\begin{aligned} \rho&= \frac{1}{4} \left[ 2\cos 2 \theta ^{\nu }_{12} - \sin 2 \theta ^{\nu }_{12} \frac{\cos ^2 \theta ^{\nu }_{13} }{\sin \theta ^{\nu }_{13} } \right] ,\end{aligned}$$

88 $$\begin{aligned} \eta&= \sin \theta ^{\nu }_{12} \left[ \cos ^2 \theta _{13} \sin \theta ^{\nu }_{12} \!-\! \cos \theta ^{\nu }_{12} \left( \! \sin \theta ^{\nu }_{13} \!-\! \frac{\sin ^2 \theta _{13}}{\sin \theta ^{\nu }_{13}} \!\right) \!\right] . \end{aligned}$$

Using Eq. (86) for $\sin ^2 \theta _{12}$ with $\rho$ and $\eta$ as given above, one can express $\sin ^2 \theta ^e_{13}$ in terms of $\theta _{12}$, $\theta _{13}$, $\theta ^{\nu }_{12}$, $\theta ^{\nu }_{13}$:

89 $$\begin{aligned} \sin ^2 \theta ^e_{13}= \frac{2 \cos ^2 \theta _{13}\sin \theta ^{\nu }_{13} (\sin ^2 \theta _{12} - \sin ^2 \theta ^{\nu }_{12}) - \sin 2 \theta ^{\nu }_{12} \sin ^2 \theta _{13} + \sin 2\theta ^{\nu }_{12} \sin ^2 \theta ^{\nu }_{13}}{\cos 2 \theta ^{\nu }_{12} \sin \theta ^{\nu }_{13} - \cos \theta ^{\nu }_{12} \sin \theta ^{\nu }_{12} \cos ^2 \theta ^{\nu }_{13}} . \end{aligned}$$

In the limit of vanishing $\theta ^{\nu }_{13}$ we find $\sin ^2 \theta ^e_{13} = 2 \sin ^2 \theta _{13}$, as obtained in Sect. 2.2.

Further, using Eq. (84), we can write $\cos \omega$ in terms of the standard parametrisation mixing angles and the known $\theta ^e_{13}$, $\theta ^{\nu }_{12}$ and $\theta ^{\nu }_{13}$:

90 $$\begin{aligned} \cos \omega&= [ 2 \cos ^2 \theta _{13} (\sin \theta ^e_{13})^{-2} (\sin \theta ^{\nu }_{12})^{-2} \sin ^2 \theta _{12}\nonumber \\&\quad - 2 \cos ^2 \theta ^{\nu }_{13} \cot ^2 \theta ^e_{13} - (\cot \theta ^{\nu }_{12} - \sin \theta ^{\nu }_{13})^2] \nonumber \\&\quad \times (\cos \theta ^{\nu }_{13})^{-1} \tan \theta ^e_{13} [ 2 \sqrt{2} (\cot \theta ^{\nu }_{12} - \sin \theta ^{\nu }_{13})]^{-1} . \end{aligned}$$

Analogously to the case considered in the preceding subsection, from the requirements $(0 < \sin ^2 \theta ^e_{13} < 1) \wedge (-1 < \cos \omega < 1)$ one can obtain for a given $\theta ^{\nu }_{13}$, each of the symmetry values of $\theta ^{\nu }_{12}$ considered and $\theta ^{\nu }_{23} = -\pi /4$ lower and upper bounds on the value of $\sin ^2 \theta _{12}$. These bounds will be discussed in Sect. 5.3.

Comparing again the imaginary parts of $U^*_{e1} U^*_{\mu 3} U_{e3} U_{\mu 1}$, obtained using Eq. (18) with $(ij) = (13)$ and $\theta ^{\nu }_{23} = -\pi /4$, and in the standard parametrisation of *U*, one gets the following relation between $\sin \omega$ and $\sin \delta$ for arbitrarily fixed $\theta ^{\nu }_{12}$ and $\theta ^{\nu }_{13}$:

91 $$\begin{aligned} \sin \delta&= -\frac{\sin 2 \theta ^e_{13}}{2 \sin 2 \theta _{12} \sin 2 \theta _{13} \cos \theta _{23}}\nonumber \\&\quad \times [ \cos ^2 \theta ^{\nu }_{13} \sin 2 \theta ^{\nu }_{12} - 2 \cos 2 \theta ^{\nu }_{12} \sin \theta ^{\nu }_{13} ] \sin \omega . \end{aligned}$$

Exploiting the equality of the expressions for $|U_{\mu 1}|$ written in the two parametrisations,

92 $$\begin{aligned} |U_{\mu 1}|= & {} |\cos \theta _{23} \sin \theta _{12} + e^{i \delta } \cos \theta _{12} \sin \theta _{13} \sin \theta _{23}|\nonumber \\= & {} \frac{1}{\sqrt{2}} |\cos \theta ^{\nu }_{12} \sin \theta ^{\nu }_{13} - \sin \theta ^{\nu }_{12}|, \end{aligned}$$

we get the following sum rule for $\cos \delta$:

93 $$\begin{aligned} \cos \delta&= -\frac{1}{\sin 2\theta _{12} \sin \theta _{13} |\cos \theta ^{\nu }_{13}| (1 \!-\! 2\sin ^2\theta _{13} \!+\! \sin ^2\theta ^{\nu }_{13})^{\frac{1}{2}}}\nonumber \\&\quad \times [(1 - 2\sin ^2\theta _{13} + \sin ^2\theta ^{\nu }_{13}) \sin ^2 \theta _{12} \nonumber \\&\quad + \cos ^2\theta _{12}\sin ^2\theta _{13}\cos ^2\theta ^{\nu }_{13} \!-\! \cos ^2\theta _{13} (\sin \theta ^{\nu }_{12} \!-\! \cos \theta ^{\nu }_{12} \nonumber \\&\quad \times \sin \theta ^{\nu }_{13})^2]. \end{aligned}$$

For $\theta ^{\nu }_{13} = 0$ this sum rule reduces to the sum rule for $\cos \delta$ given in Eq. (27).

In the parametrisation of the PMNS matrix considered in this subsection, the $J_\mathrm{CP}$ factor reads

94 $$\begin{aligned} J_\mathrm{CP}= & {} -\frac{1}{8 \sqrt{2}} \sin \omega \cos \theta ^{\nu }_{13} \sin 2\theta ^e_{13}\nonumber \\&\times [\cos ^2 \theta ^{\nu }_{13} \sin 2\theta ^{\nu }_{12} -\, 2 \sin \theta ^{\nu }_{13} \cos 2\theta ^{\nu }_{12}] . \end{aligned}$$

In the case of an arbitrary fixed value of $\theta ^{\nu }_{23}$, as it is not difficult to show, we have

95 $$\begin{aligned} \sin ^2 \theta _{23}&= \frac{|U_{\mu 3}|^2}{1-|U_{e3}|^2} = \frac{\sin ^2 \theta ^{\nu }_{23} \cos ^2 \theta ^{\nu }_{13}}{1 - \sin ^2 \theta _{13}} , \end{aligned}$$

and

96 $$\begin{aligned} |U_{\mu 1}|= & {} |\cos \theta _{23} \sin \theta _{12} + e^{i \delta } \cos \theta _{12} \sin \theta _{13} \sin \theta _{23}|\nonumber \\= & {} |\cos \theta ^{\nu }_{12} \sin \theta ^{\nu }_{13} \sin \theta ^{\nu }_{23} + \sin \theta ^{\nu }_{12} \cos \theta ^{\nu }_{23}|. \end{aligned}$$

Using Eqs. (95) and (96), we obtain in this case

97 $$\begin{aligned}&\cos \delta \!=\! -\frac{1}{\sin 2 \theta _{12} \sin \theta _{13} | \cos \theta ^{\nu }_{13} \sin \theta ^{\nu }_{23}| (\cos ^2 \theta _{13} \!-\! \cos ^2 \theta ^{\nu }_{13} \sin ^2 \theta ^{\nu }_{23})^{\frac{1}{2}}} \nonumber \\&\quad \qquad \quad \times [ (\cos ^2 \theta _{13} - \cos ^2 \theta ^{\nu }_{13} \sin ^2 \theta ^{\nu }_{23}) \sin ^2 \theta _{12}\nonumber \\&\quad \qquad \quad + \cos ^2 \theta _{12} \sin ^2 \theta _{13} \cos ^2 \theta ^{\nu }_{13} \sin ^2 \theta ^{\nu }_{23} \nonumber \\&\quad \qquad \quad - \cos ^2 \theta _{13} ( \cos \theta ^{\nu }_{12} \sin \theta ^{\nu }_{13} \sin \theta ^{\nu }_{23} + \sin \theta ^{\nu }_{12} \cos \theta ^{\nu }_{23})^2 ] . \end{aligned}$$

The sum rules derived in Sects. 2–4 and corresponding to arbitrary fixed values of the angles contained in the matrix $\tilde{U}_{\nu }$, Eqs. (13), (21), (32), (45), (63), (81) and (97), are summarised in Table 1. In Table 2 we give the corresponding formulae for $\sin ^2 \theta _{23}$.

**Table 1:** Summary of the sum rules for $\cos \delta$. The parameter $\kappa$ is defined in Sect. 3.2 after Eq. (58). The sum rule corresponding to the parametrisation of *U*, $R_{12}(\theta ^e_{12})R_{23}(\theta ^e_{23})\Psi R_{23}(\theta ^{\nu }_{23})R_{12}(\theta ^{\nu }_{12})Q_0$, is the one quoted in Eq. (13) and was derived in [45]

Parametrisation of *U*	$\cos \delta$
$R_{12}(\theta ^e_{12}) \, \Psi \, R_{23}(\theta ^{\nu }_{23}) \, R_{12}(\theta ^{\nu }_{12}) \, Q_0$	$\dfrac{(\cos 2 \theta _{13} - \cos 2 \theta ^{\nu }_{23})^{\frac{1}{2}}}{\sqrt{2}\sin 2\theta _{12}\sin \theta _{13}|\cos \theta ^{\nu }_{23}|}\, \bigg [ \cos 2\theta ^{\nu }_{12} + \left( \sin ^2\theta _{12} - \cos ^2\theta ^{\nu }_{12} \right) \, \dfrac{2\sin ^2 \theta ^{\nu }_{23}-(3+\cos 2 \theta ^{\nu }_{23}) \sin ^2\theta _{13}}{\cos 2 \theta _{13} - \cos 2 \theta ^{\nu }_{23}} \bigg ]$
$R_{13}(\theta ^e_{13}) \, \Psi \, R_{23}(\theta ^{\nu }_{23}) \, R_{12}(\theta ^{\nu }_{12}) \, Q_0$	$-\dfrac{(\cos 2 \theta _{13} + \cos 2 \theta ^{\nu }_{23})^{\frac{1}{2}}}{\sqrt{2}\sin 2\theta _{12}\sin \theta _{13}|\sin \theta ^{\nu }_{23}|}\, \bigg [ \cos 2\theta ^{\nu }_{12} + \left( \sin ^2\theta _{12} - \cos ^2\theta ^{\nu }_{12} \right) \, \dfrac{2\cos ^2 \theta ^{\nu }_{23}-(3-\cos 2 \theta ^{\nu }_{23}) \sin ^2\theta _{13}}{\cos 2 \theta _{13} + \cos 2 \theta ^{\nu }_{23}} \bigg ]$
$R_{12}(\theta ^e_{12}) \, R_{23}(\theta ^e_{23}) \, \Psi \, R_{23}(\theta ^{\nu }_{23}) \, R_{12}(\theta ^{\nu }_{12}) \, Q_0$	$\dfrac{\tan \theta _{23}}{\sin 2\theta _{12}\sin \theta _{13}}\, [\cos 2\theta ^{\nu }_{12} + (\sin ^2\theta _{12} - \cos ^2\theta ^{\nu }_{12} )\, (1 - \cot ^2\theta _{23}\,\sin ^2\theta _{13} ) ]$
$R_{13}(\theta ^e_{13}) \, R_{23}(\theta ^e_{23}) \, \Psi \, R_{23}(\theta ^{\nu }_{23}) \, R_{12}(\theta ^{\nu }_{12}) \, Q_0$	$-\dfrac{\cot \theta _{23}}{\sin 2\theta _{12}\sin \theta _{13}}\, [\cos 2\theta ^{\nu }_{12} + (\sin ^2\theta _{12} - \cos ^2\theta ^{\nu }_{12} )\, (1 - \tan ^2\theta _{23}\,\sin ^2\theta _{13}) ]$
$R_{12}(\theta ^e_{12}) \, R_{13}(\theta ^e_{13}) \, \Psi \, R_{23}(\theta ^{\nu }_{23}) \, R_{12}(\theta ^{\nu }_{12}) \, Q_0$	$\dfrac{1}{\sin 2 \theta _{12} \sin 2 \theta _{23} \sin \theta _{13}} \bigg [ \dfrac{2 \kappa \cos \omega \sin 2 \theta ^{\nu }_{12} \sin \theta ^{\nu }_{23} \cos \theta _{13} \cos \theta _{23}}{\cos ^2 \theta ^{\nu }_{23}} (\cos ^2 \theta ^{\nu }_{23} - \cos ^2 \theta _{13} \cos ^2 \theta _{23})^{\frac{1}{2}}$
	$$\quad - \cos 2 \theta ^{\nu }_{12} \Bigg (1 - \dfrac{\cos ^2 \theta _{13} \cos ^2 \theta _{23}}{\cos ^2 \theta ^{\nu }_{23} }(\sin ^2 \theta ^{\nu }_{23} + 1) \Bigg ) +\cos 2 \theta _{12} (\cos ^2 \theta _{23} \sin ^2 \theta _{13} - \sin ^2 \theta _{23} ) \bigg ]$$
$R_{12}(\theta ^e_{12}) \, \Psi \, R_{23}(\theta ^{\nu }_{23})\, R_{13}(\theta ^{\nu }_{13}) \, R_{12}(\theta ^{\nu }_{12}) \, Q_0$	$\dfrac{1}{\sin 2 \theta _{12} \sin \theta _{13} | \cos \theta ^{\nu }_{13} \cos \theta ^{\nu }_{23}| (\cos ^2 \theta _{13} - \cos ^2 \theta ^{\nu }_{13} \cos ^2 \theta ^{\nu }_{23})^{\frac{1}{2}}}[ (\cos ^2 \theta _{13} - \cos ^2 \theta ^{\nu }_{13} \cos ^2 \theta ^{\nu }_{23}) \sin ^2 \theta _{12}$
	$$\quad + \cos ^2 \theta _{12} \sin ^2 \theta _{13} \cos ^2 \theta ^{\nu }_{13} \cos ^2 \theta ^{\nu }_{23} - \cos ^2 \theta _{13} ( \cos \theta ^{\nu }_{12} \sin \theta ^{\nu }_{13} \cos \theta ^{\nu }_{23} - \sin \theta ^{\nu }_{12} \sin \theta ^{\nu }_{23})^2 ] $$
$R_{13}(\theta ^e_{13}) \, \Psi \, R_{23}(\theta ^{\nu }_{23})\, R_{13}(\theta ^{\nu }_{13}) \, R_{12}(\theta ^{\nu }_{12}) \, Q_0$	$-\dfrac{1}{\sin 2 \theta _{12} \sin \theta _{13} | \cos \theta ^{\nu }_{13} \sin \theta ^{\nu }_{23}| (\cos ^2 \theta _{13} - \cos ^2 \theta ^{\nu }_{13} \sin ^2 \theta ^{\nu }_{23})^{\frac{1}{2}}} [ (\cos ^2 \theta _{13} - \cos ^2 \theta ^{\nu }_{13} \sin ^2 \theta ^{\nu }_{23}) \sin ^2 \theta _{12}$
	$$+ \cos ^2 \theta _{12} \sin ^2 \theta _{13} \cos ^2 \theta ^{\nu }_{13} \sin ^2 \theta ^{\nu }_{23} - \cos ^2 \theta _{13} ( \cos \theta ^{\nu }_{12} \sin \theta ^{\nu }_{13} \sin \theta ^{\nu }_{23} + \sin \theta ^{\nu }_{12} \cos \theta ^{\nu }_{23})^2 ]$$

**Table 2:** Summary of the formulae for $\sin ^2 \theta _{23}$. The formula for $\sin ^2 \hat{\theta }_{23}$ is given in Eq. (35)

Parametrisation of *U*	$\sin ^2 \theta _{23}$
$R_{12}(\theta ^e_{12}) \, \Psi \, R_{23}(\theta ^{\nu }_{23}) \, R_{12}(\theta ^{\nu }_{12}) \, Q_0$	$\dfrac{\sin ^2 \theta ^{\nu }_{23}-\sin ^2 \theta _{13}}{1 - \sin ^2 \theta _{13}}$
$R_{13}(\theta ^e_{13}) \, \Psi \, R_{23}(\theta ^{\nu }_{23}) \, R_{12}(\theta ^{\nu }_{12}) \, Q_0$	$\dfrac{\sin ^2 \theta ^{\nu }_{23}}{1 - \sin ^2 \theta _{13}}$
$R_{12}(\theta ^e_{12}) \, R_{23}(\theta ^e_{23}) \, \Psi \, R_{23}(\theta ^{\nu }_{23}) \, R_{12}(\theta ^{\nu }_{12}) \, Q_0$	$\dfrac{\sin ^2 \hat{\theta }_{23} - \sin ^2 \theta _{13}}{1 - \sin ^2 \theta _{13}}$
$R_{13}(\theta ^e_{13}) \, R_{23}(\theta ^e_{23}) \, \Psi \, R_{23}(\theta ^{\nu }_{23}) \, R_{12}(\theta ^{\nu }_{12}) \, Q_0$	$\dfrac{\sin ^2 \hat{\theta }_{23}}{1 - \sin ^2 \theta _{13}}$
$R_{12}(\theta ^e_{12}) \, R_{13}(\theta ^e_{13}) \, \Psi \, R_{23}(\theta ^{\nu }_{23}) \, R_{12}(\theta ^{\nu }_{12}) \, Q_0$	$\dfrac{\sin ^2 \theta ^{\nu }_{23} - \sin ^2 \theta _{13} + \sin ^2 \theta ^e_{13} \cos ^2 \theta ^{\nu }_{23}}{1 - \sin ^2 \theta _{13}}$
$R_{12}(\theta ^e_{12}) \, \Psi \, R_{23}(\theta ^{\nu }_{23})\, R_{13}(\theta ^{\nu }_{13}) \, R_{12}(\theta ^{\nu }_{12}) \, Q_0$	$1 - \dfrac{\cos ^2 \theta ^{\nu }_{23} \cos ^2 \theta ^{\nu }_{13}}{1 - \sin ^2 \theta _{13}}$
$R_{13}(\theta ^e_{13}) \, \Psi \, R_{23}(\theta ^{\nu }_{23})\, R_{13}(\theta ^{\nu }_{13}) \, R_{12}(\theta ^{\nu }_{12}) \, Q_0$	$\dfrac{\sin ^2 \theta ^{\nu }_{23} \cos ^2 \theta ^{\nu }_{13}}{1 - \sin ^2 \theta _{13}}$

## Predictions

In this section we present results of a statistical analysis, performed using the procedure described in Appendix A (see also [49, 50]), which allows us to get the dependence of the $\chi ^2$ function on the value of $\delta$ and on the value of the $J_\mathrm{CP}$ factor. In what follows we always assume that $\theta ^{\nu }_{23} = -\pi /4$. We find that in the case corresponding to Eq. (16) with $(ij) = (12)$, analysed in [45], the results for $\chi ^2$ as a function of $\delta$ or $J_\mathrm{CP}$ are rather similar to those obtained in [49, 50] in the case of the parametrisation defined by Eq. (17) with (*ij*) –$(kl) = (12)$– (23). The main difference between these two cases is the predictions for $\sin ^2 \theta _{23}$, which can deviate only by approximately $0.5 \sin ^2 \theta _{13}$ from 0.5 in the first case and by a significantly larger amount in the second. As a consequence, the predictions in the first case are somewhat less favoured by the current data than in the second case, which is reflected in the higher value of $\chi ^2$ at the minimum, $\chi ^2_\mathrm{min}$. Similar conclusions hold on comparing the results in the case of $\theta ^e_{13}-(\theta ^\nu _{23},\theta ^\nu _{12})$ rotations, described in Sect. 2.2, and in the corresponding case defined by Eq. (17) with (*ij*) –$(kl) = (13)$– (23) and discussed in Sect. 3.1. Therefore, in what concerns these four schemes, in what follows we will present results of the statistical analysis of the predictions for $\delta$ and the $J_\mathrm{CP}$ factor only for the scheme with $(\theta ^e_{13},\theta ^e_{23})$–$(\theta ^\nu _{23},\theta ^\nu _{12})$ rotations, considered in Sect. 3.1.

We show in Tables 3 and 4 the predictions for $\cos \delta$ and $\delta$ for all the schemes considered in the present study using the current best fit values of the neutrino mixing parameters $\sin ^2\theta _{12}$, $\sin ^2\theta _{23}$ and $\sin ^2\theta _{13}$, quoted in Eqs. (3)–(5), which enter into the sum rule expressions for $\cos \delta$, Eqs. (13), (27), (45), (58), (77), (93) and Eq. (50) in Ref. [45], unless other values of the indicated mixing parameters are explicitly specified. We present results only for the NO neutrino mass spectrum, since the results for the IO spectrum differ insignificantly. Several comments are in order.

**Table 3:** The predicted values of $\cos \delta$ using the current best fit values of the mixing angles, quoted in Eqs. (3)–(5) and corresponding to neutrino mass spectrum with NO, except for the case $(\theta ^e_{12},\theta ^e_{13})$–$(\theta ^\nu _{23}, \theta ^\nu _{12})$ with $\omega = 0$ and $\kappa = 1$, in which $\sin ^2 \theta _{23} = 0.48802$ is used. We have defined $a = \sin ^{-1} (1 / 3)$, $b = \sin ^{-1} (1 / \sqrt{2 + r})$, $c = \sin ^{-1} (1 / \sqrt{3})$ and $d = \sin ^{-1} (\sqrt{3 - r}/2)$. For the last two schemes we give in square brackets the values of $[\theta ^\nu _{13}, \theta ^\nu _{12}]$. TBM, GRA, GRB, HG and BM (LC) refer, in particular, to the different fixed values of $\theta ^{\nu }_{12} = c, b, d, \pi /6$ and $\pi /4$, respectively. See text for further details

Scheme	TBM	GRA	GRB	HG	BM (LC)
$\theta ^e_{12}-(\theta ^\nu _{23}, \theta ^\nu _{12})$	$-0.114$	0.289	$-0.200$	0.476	–
$\theta ^e_{13}-(\theta ^\nu _{23}, \theta ^\nu _{12})$	0.114	$-0.289$	0.200	$-0.476$	–
$(\theta ^e_{12},\theta ^e_{23})-(\theta ^\nu _{23}, \theta ^\nu _{12})$	$-0.091$	0.275	$-0.169$	0.445	–
$(\theta ^e_{13},\theta ^e_{23})-(\theta ^\nu _{23}, \theta ^\nu _{12})$	0.151	$-0.315$	0.251	$-0.531$	–
$(\theta ^e_{12},\theta ^e_{13})-(\theta ^\nu _{23}, \theta ^\nu _{12})$	$-0.122$	0.282	$-0.208$	0.469	–

Scheme	$[\pi /20,-\pi /4]$	$[\pi /10,-\pi /4]$	$[a,-\pi /4]$	$[\pi /20,b]$	$[\pi /20,\pi /6]$
$\theta ^e_{12}-(\theta ^\nu _{23}, \theta ^\nu _{13}, \theta ^\nu _{12})$	$-0.222$	0.760	0.911	$-0.775$	$-0.562$

Scheme	$[\pi /20,c]$	$[\pi /20,\pi /4]$	$[\pi /10,\pi /4]$	$[a,\pi /4]$	$[\pi /20,d]$
$\theta ^e_{13}-(\theta ^\nu _{23}, \theta ^\nu _{13}, \theta ^\nu _{12})$	$-0.866$	0.222	$-0.760$	$-0.911$	$-0.791$

**Table 4:** The same as in Table 3, but for $\delta$ given in degrees (see text for further details)

Scheme	TBM	GRA	GRB	HG	BM (LC)
$\theta ^e_{12}-(\theta ^\nu _{23}, \theta ^\nu _{12})$	$97 \vee 263$	$73 \vee 287$	$102 \vee 258$	$62 \vee 298$	–
$\theta ^e_{13}-(\theta ^\nu _{23}, \theta ^\nu _{12})$	$83 \vee 277$	$107 \vee 253$	$78 \vee 282$	$118 \vee 242$	–
$(\theta ^e_{12},\theta ^e_{23})-(\theta ^\nu _{23}, \theta ^\nu _{12})$	$95 \vee 265$	$74 \vee 286$	$100 \vee 260$	$64 \vee 296$	–
$(\theta ^e_{13},\theta ^e_{23})-(\theta ^\nu _{23}, \theta ^\nu _{12})$	$81 \vee 279$	$108 \vee 252$	$75 \vee 285$	$122 \vee 238$	–
$(\theta ^e_{12},\theta ^e_{13})-(\theta ^\nu _{23}, \theta ^\nu _{12})$	$97 \vee 263$	$74 \vee 286$	$102 \vee 258$	$62 \vee 298$	–

Scheme	$[\pi /20,-\pi /4]$	$[\pi /10,-\pi /4]$	$[a,-\pi /4]$	$[\pi /20,b]$	$[\pi /20,\pi /6]$
$\theta ^e_{12}-(\theta ^\nu _{23}, \theta ^\nu _{13}, \theta ^\nu _{12})$	$103 \vee 257$	$41 \vee 319$	$24 \vee 336$	$141 \vee 219$	$124 \vee 236$

Scheme	$[\pi /20,c]$	$[\pi /20,\pi /4]$	$[\pi /10,\pi /4]$	$[a,\pi /4]$	$[\pi /20,d]$
$\theta ^e_{13}-(\theta ^\nu _{23}, \theta ^\nu _{13}, \theta ^\nu _{12})$	$150 \vee 210$	$77 \vee 283$	$139 \vee 221$	$156 \vee 204$	$142 \vee 218$

We do not present predictions for the BM (LC) symmetry form of $\tilde{U}_{\nu }$ in Tables 3 and 4, because for the current best fit values of $\sin ^2 \theta _{12}$, $\sin ^2 \theta _{23}$, $\sin ^2 \theta _{13}$ the corresponding sum rules give unphysical values of $\cos \delta$ (see, however, Refs. [45, 49, 50]). Using the best fit value of $\sin ^2 \theta _{13}$, we get physical values of $\cos \delta$ in the BM case for the following minimal values of $\sin ^2 \theta _{12}$:

$$\begin{aligned} \cos \delta&= -0.993 (\delta \cong \pi ) \,\mathrm{for}\, \sin ^2 \theta _{12}\\&= 0.348 \,\mathrm{in \, the \, scheme}\, \theta ^e_{12} - (\theta ^\nu _{23}, \theta ^\nu _{12}), \\ \cos \delta&= +0.993 (\delta \cong 0)\, \mathrm{for}\, \sin ^2 \theta _{12}\\&= 0.348 \,\mathrm{in \, the \, scheme}\, \theta ^e_{13} - (\theta ^\nu _{23}, \theta ^\nu _{12}), \\ \cos \delta&= -0.994 (\delta \cong \pi )\, \mathrm{for}\, \sin ^2 \theta _{12}\\&= 0.349 \,\mathrm{in \, the \, scheme}\, (\theta ^e_{12},\theta ^e_{13}) - (\theta ^\nu _{23}, \theta ^\nu _{12}),\\ \cos \delta&= -0.996 (\delta \cong \pi )\, \mathrm{for}\, \sin ^2 \theta _{12}\\&= 0.332\, \mathrm{in \, the \, scheme}\, (\theta ^e_{12},\theta ^e_{23}) - (\theta ^\nu _{23}, \theta ^\nu _{12}), \\ \cos \delta&= +0.997 (\delta \cong 0)\, \mathrm{for}\, \sin ^2 \theta _{12}\\&= 0.368\, \mathrm{in \, the \, scheme}\, (\theta ^e_{13},\theta ^e_{23}) - (\theta ^\nu _{23}, \theta ^\nu _{12}), \end{aligned}$$

where in the case of the scheme $(\theta ^e_{12},\theta ^e_{13}) - (\theta ^\nu _{23}, \theta ^\nu _{12})$ we fixed $\sin ^2 \theta _{23} = 0.48802$ (we will comment later on this choice), while $\sin ^2 \theta _{23}$ was set to its best fit value for the last two set-ups.

Results for the scheme $(\theta ^e_{12},\theta ^e_{23}) - (\theta ^\nu _{23}, \theta ^\nu _{12})$ in the cases of the TBM and BM symmetry forms of the matrix $\tilde{U}_{\nu }$ were presented first in [46], while results for the same scheme and the GRA, GRB and HG symmetry forms of $\tilde{U}_{\nu }$, as well as for the scheme $\theta ^e_{12}-(\theta ^\nu _{23}, \theta ^\nu _{12})$ for all symmetry forms considered, were obtained first in [45]. The predictions for $\cos \delta$ and $\delta$ were derived in [45, 46] for the best fit values of the relevant neutrino mixing parameters found in an earlier global analysis performed in [47] and differ somewhat (albeit not much) from those quoted in Tables 3 and 4. The values under discussion given in these tables are from [49] and correspond to the best fit values quoted in Eqs. (3)–(5).

The predictions for $\cos \delta$ of the $\theta ^e_{12} - (\theta ^\nu _{23}, \theta ^\nu _{12})$ and $\theta ^e_{13} - (\theta ^\nu _{23}, \theta ^\nu _{12})$ schemes for each of the symmetry forms of $\tilde{U}_{\nu }$ considered differ only by sign. The $\theta ^e_{12}-(\theta ^\nu _{23}, \theta ^\nu _{12})$ scheme and the $(\theta ^e_{12},\theta ^e_{13})$–$(\theta ^\nu _{23}, \theta ^\nu _{12})$ scheme with $\omega = 0$ provide very similar predictions for $\cos \delta$.

In the schemes with three rotations in $\tilde{U}_{\nu }$ we consider, $\cos \delta$ has values which differ significantly (being larger in absolute value) from the values predicted by the schemes with two rotations in $\tilde{U}_{\nu }$ discussed by us, the only exceptions being (i) the $\theta ^e_{12(13)}-(\theta ^\nu _{23}, \theta ^\nu _{13}, \theta ^\nu _{12})$ scheme with $[\theta ^\nu _{13},\theta ^\nu _{12}] =[\pi /20,^{~-~}_{(+)}\pi /4]$, for which $|\cos \delta | = 0.222$, and (ii) $\theta ^e_{12} - (\theta ^\nu _{23}, \theta ^\nu _{13}, \theta ^\nu _{12})$ scheme with $[\theta ^\nu _{13},\theta ^\nu _{12}] =[\pi /20,\pi /6]$ in which $\cos \delta = -\,0.562$.

The predictions for $\cos \delta$ of the schemes denoted as $(\theta ^e_{12},\theta ^e_{23})$–$(\theta ^\nu _{23}, \theta ^\nu _{12})$ and $(\theta ^e_{13},\theta ^e_{23})$–$(\theta ^\nu _{23}, \theta ^\nu _{12})$ differ for each of the symmetry forms of $\tilde{U}_{\nu }$ considered both by sign and magnitude. If the best fit value of $\theta _{23}$ were $\pi /4$, these predictions would differ only by sign.

In the case of the $(\theta ^e_{12},\theta ^e_{13}) - (\theta ^\nu _{23}, \theta ^\nu _{12})$ scheme with $\omega = 0$, the predictions for $\cos \delta$ are very sensitive to the value of $\sin ^2 \theta _{23}$. Using the best fit values of $\sin ^2 \theta _{12}$ and $\sin ^2 \theta _{13}$ for the NO neutrino mass spectrum, quoted in Eqs. (3) and (5), we find from the constraints $(-1 < \cos \psi < 1)$ and $(0 < \sin ^2 \theta ^e_{13} < 1) \wedge (0 < \sin ^2 \theta ^e_{12} < 1)$, where $\sin ^2 \theta ^e_{13}$, $\sin ^2 \theta ^e_{12}$ and $\cos \psi$ are given in Eqs. (53)–(55), that $\sin ^2 \theta _{23}$ should lie in the following intervals:

$$\begin{aligned}&(0.488,0.496) \cup (0.847,0.909) \quad \text{ for } \text{ TBM }; \\&(0.488,0.519) \cup (0.948,0.971) \quad \text{ for } \text{ BM }; \\&(0.488,0.497) \cup (0.807,0.880) \quad \text{ for } \text{ GRA }; \\&(0.488,0.498) \cup (0.856,0.914) \quad \text{ for } \text{ GRB }; \\&(0.488,0.500) \cup (0.787,0.866) \quad \text{ for } \text{ HG }. \end{aligned}$$

Obviously, the quoted intervals with $\sin ^2 \theta _{23} \ge 0.78$ are ruled out by the current data. We observe that a small increase of $\sin ^2 \theta _{23}$ from the value 0.48802^9^ produces a relatively large variation of $\cos \delta$. The strong dependence of $\cos \delta$ on $\sin ^2 \theta _{23}$ takes place for values of $\omega$ satisfying roughly $\cos \omega \gtrsim 0.01$. In contrast, for $\cos \omega = 0$, $\cos \delta$ exhibits a relatively weak dependence on $\sin ^2 \theta _{23}$. For the reasons related to the dependence of $\cos \delta$ on $\omega$ we are not going to present results of the statistical analysis in this case. This can be done in specific models of neutrino mixing, in which the value of the phase $\omega$ is fixed by the model.

**Fig. 1:**
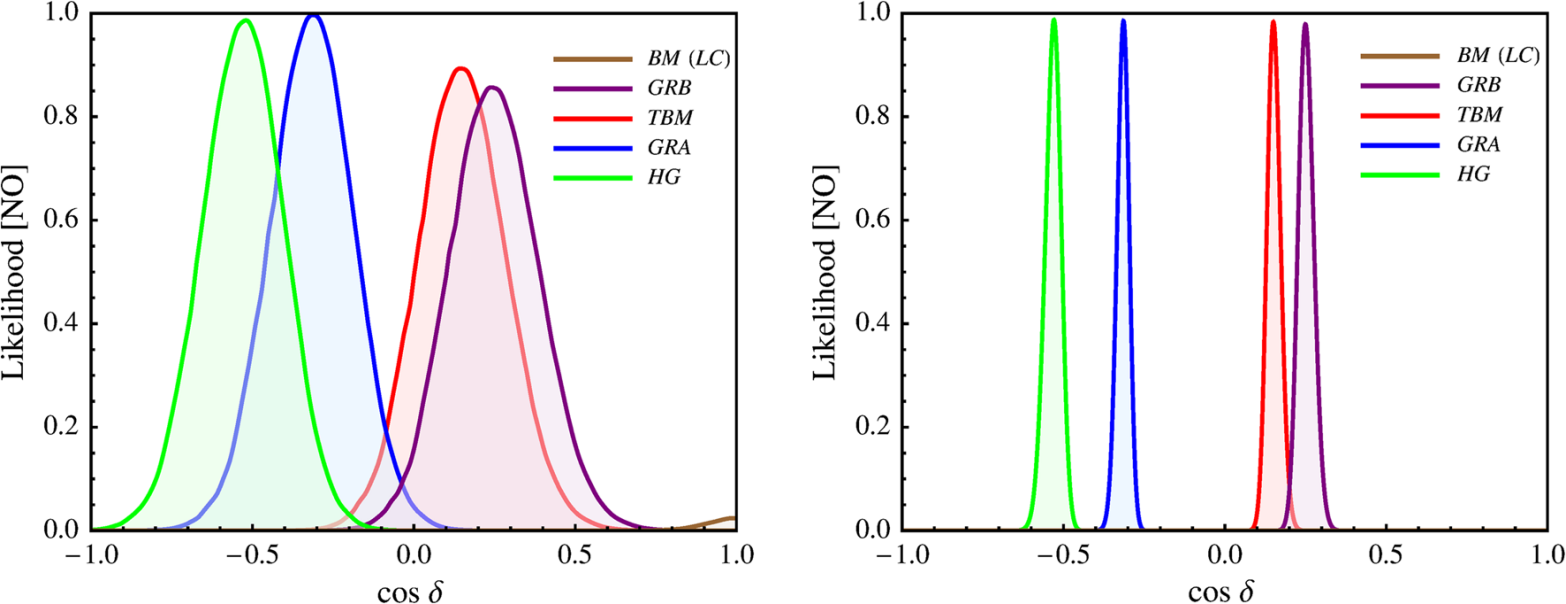
The likelihood function versus $\cos \delta$ for the NO neutrino mass spectrum after marginalising over $\sin ^2\theta _{13}$ and $\sin ^2 \theta _{23}$ for the TBM, BM (LC), GRA, GRB and HG symmetry forms of the matrix $\tilde{U}_{\nu }$ in the $(\theta ^e_{13},\theta ^e_{23}) - (\theta ^\nu _{23}, \theta ^\nu _{12})$ set-up. The results shown are obtained using Eq. (45) and (i) the latest results on the mixing parameters $\sin ^2\theta _{12}$, $\sin ^2\theta _{13}$, $\sin ^2\theta _{23}$ and $\delta$ found in the global analysis of the neutrino oscillation data [23] (*left panel*), and (ii) the prospective $1\sigma$ uncertainties on $\sin ^2\theta _{12}$, $\sin ^2\theta _{13}$, $\sin ^2\theta _{23}$ and the Gaussian approximation for the likelihood function (*right panel*) (see text for further details)

### The scheme with $(\theta ^e_{13},\theta ^e_{23})-(\theta ^\nu _{23}, \theta ^\nu _{12})$ rotations

In the left panel of Fig. 1 we show the likelihood function, defined as

98 $$\begin{aligned} L(\cos \delta ) \propto \exp \left( - \frac{\chi ^2 (\cos \delta )}{2} \right) , \end{aligned}$$

versus $\cos \delta$ for the NO neutrino mass spectrum for the scheme with $(\theta ^e_{13},\theta ^e_{23})$–$(\theta ^\nu _{23}, \theta ^\nu _{12})$ rotations.^10^ This function represents the most probable values of $\cos \delta$ for each of the symmetry forms considered. In the analysis performed by us we use as input the current global neutrino oscillation data on $\sin ^2 \theta _{12}$, $\sin ^2 \theta _{23}$, $\sin ^2 \theta _{13}$ and $\delta$ [23]. The maxima of $L(\cos \delta )$, $L(\chi ^2 = \chi ^2_\mathrm{min})$, for the different symmetry forms of $\tilde{U}_{\nu }$ considered, correspond to the values of $\cos \delta$ given in Table 3. The results shown are obtained by marginalising over $\sin ^2 \theta _{13}$ and $\sin ^2 \theta _{23}$ for a fixed value of $\delta$ (for details of the statistical analysis see Appendix A and [49, 50]). The $n \sigma$ confidence level (CL) region corresponds to the interval of values of $\cos \delta$ for which $L(\cos \delta ) \ge L(\chi ^2 = \chi ^2_\mathrm{min}) \cdot L(\chi ^2 = n^2)$. Here $\chi ^2_\mathrm{min}$ is the value of $\chi ^2$ in the minimum.

As can be observed from the left panel of Fig. 1, for the TBM and GRB forms there is a substantial overlap of the corresponding likelihood functions. The same observation holds also for the GRA and HG forms. However, the likelihood functions of these two sets of symmetry forms overlap only at $3\sigma$ and in a small interval of values of $\cos \delta$. Thus, the TBM/GRB, GRA/HG and BM (LC) symmetry forms might be distinguished with a not very demanding (in terms of precision) measurement of $\cos \delta$. At the maximum, the non-normalised likelihood function equals $\exp ( - \chi ^2_\mathrm{min}/2)$, and this value allows one to judge quantitatively about the compatibility of a given symmetry form with the global neutrino oscillation data, as we have pointed out.

In the right panel of Fig. 1 we present *L* versus $\cos \delta$ within the Gaussian approximation (see [49, 50] for details), using the current best fit values of $\sin ^2 \theta _{12}$, $\sin ^2 \theta _{23}$, $\sin ^2 \theta _{13}$ for the NO spectrum, given in Eqs. (3)–(5), and the prospective $1\sigma$ uncertainties in the measurement of these mixing parameters. More specifically, we use as $1\sigma$ uncertainties (i) 0.7 % for $\sin ^2 \theta _{12}$, which is the prospective sensitivity of the JUNO experiment [69], (ii) 5 % for $\sin ^2 \theta _{23}$,^11^ obtained from the prospective uncertainty of 2 % [4] on $\sin ^2 2 \theta _{23}$ expected to be reached in the NOvA and T2K experiments, and (iii) 3 % for $\sin ^2 \theta _{13}$, deduced from the error of 3 % on $\sin ^2 2 \theta _{13}$ planned to be reached in the Daya Bay experiment [4, 71]. The BM (LC) case is quite sensitive to the values of $\sin ^2 \theta _{12}$ and $\sin ^2 \theta _{23}$ and for the current best fit values is disfavoured at more than $2\sigma$.

That the BM (LC) case is disfavoured by the current data can be understood, in particular, from the following observation. Using the best fit values of $\sin ^2 \theta _{13}$ and $\sin ^2 \theta _{12}$ as well as the constraint $-1 \le \cos \alpha \le 1$, where $\cos \alpha$ is defined in Eq. (42), one finds that $\sin ^2 \theta _{23}$ should satisfy $\sin ^2 \theta _{23} \ge 0.63$, which practically coincides with the currently allowed maximal value of $\sin ^2 \theta _{23}$ at $3\sigma$ (see Eq. (4)).

It is interesting to compare the results described above and obtained in the scheme denoted by $(\theta ^e_{13},\theta ^e_{23}) - (\theta ^\nu _{23}, \theta ^\nu _{12})$ with those obtained in [49, 50] in the $(\theta ^e_{12},\theta ^e_{23}) - (\theta ^\nu _{23}, \theta ^\nu _{12})$ set-up. We recall that for each of the symmetry forms we have considered—TBM, BM, GRA, GRB and HG—$\theta ^\nu _{12}$ has a specific fixed value and $\theta ^\nu _{23} = -\pi /4$. The first thing to note is that for a given symmetry form, $\cos \delta$ is predicted to have opposite signs in the two schemes. In the scheme $(\theta ^e_{13},\theta ^e_{23})$–$(\theta ^\nu _{23}, \theta ^\nu _{12})$ analysed in the present article, one has $\cos \delta > 0$ in the TBM, GRB and BM (LC) cases, while $\cos \delta <0$ in the cases of the GRA and HG symmetry forms. As in the $(\theta ^e_{12},\theta ^e_{23})$–$(\theta ^\nu _{23}, \theta ^\nu _{12})$ set-up, there are significant overlaps between the TBM/GRB and GRA/HG forms of $\tilde{U}_{\nu }$, respectively. The BM (LC) case is disfavoured at more than $2\sigma$ confidence level. It is also important to notice that due to the fact that the best fit value of $\sin ^2\theta _{23} < 0.5$, the predictions for $\cos \delta$ for each symmetry form, obtained in the two set-ups differ not only by sign but also in absolute value, as was already pointed out in Sect. 3.1. Thus, a precise measurement of $\cos \delta$ would allow one to distinguish not only between the symmetry forms of $\tilde{U}_{\nu }$, but also could provide an indication about the structure of the matrix $\tilde{U}_e$.

We note that the predictions for $\sin ^2 \theta _{23}$ are rather similar in the cases of the two schemes discussed, $(\theta ^e_{13},\theta ^e_{23})$–$(\theta ^\nu _{23}, \theta ^\nu _{12})$ and $(\theta ^e_{12},\theta ^e_{23})$–$(\theta ^\nu _{23}, \theta ^\nu _{12})$. We give for completeness $N_{\sigma } \equiv \sqrt{\chi ^2}$ as a function of $\sin ^2 \theta _{23}$ in Appendix B.

For the rephasing invariant $J_\mathrm{CP}$, using the current global neutrino oscillation data, we find for the symmetry forms considered the following best fit values and the $3\sigma$ ranges for the NO neutrino mass spectrum:

99 $$\begin{aligned}&J_\mathrm{CP}=-0.033, -0.039 \le J_\mathrm{CP}\le -0.026,~\nonumber \\&\quad 0.030 \le J_\mathrm{CP}\le 0.036 \quad \mathrm{for \, TBM};\end{aligned}$$

100 $$\begin{aligned}&J_\mathrm{CP}= -0.004, -0.026 \le J_\mathrm{CP}\le 0.023 \quad \mathrm{for \, BM \,(LC)} ;\end{aligned}$$

101 $$\begin{aligned}&J_\mathrm{CP}=-0.032, -0.037 \le J_\mathrm{CP}\le -0.024,~\nonumber \\&\quad 0.029 \le J_\mathrm{CP}\le 0.035 \quad \mathrm{for \, GRA};\end{aligned}$$

102 $$\begin{aligned}&J_\mathrm{CP} =-0.033, -0.039 \le J_\mathrm{CP}\le -0.023,~\nonumber \\&\quad 0.028 \le J_\mathrm{CP}\le 0.036 \quad \mathrm{for \, GRB};\end{aligned}$$

103 $$\begin{aligned}&J_\mathrm{CP}=-0.028, -0.035 \le J_\mathrm{CP}\le -0.014,\nonumber \\&\quad 0.021 \le J_\mathrm{CP}\le 0.032 \quad \mathrm{for \, HG}. \end{aligned}$$

Thus, relatively large CP-violating effects in neutrino oscillations are predicted for all symmetry forms considered, the only exception being the case of the BM symmetry form.

### The scheme with $\theta ^e_{12}-(\theta ^\nu _{23}, \theta ^\nu _{13}, \theta ^\nu _{12})$ rotations

For the scheme with $\theta ^e_{12}-(\theta ^\nu _{23}, \theta ^\nu _{13}, \theta ^\nu _{12})$ rotations we find that only for particular values of $\theta ^{\nu }_{12}$ and $\theta ^{\nu }_{13}$, among those considered by us, the allowed intervals of values of $\sin ^2\theta _{12}$ satisfy the requirement that they contain in addition to the best fit value of $\sin ^2 \theta _{12}$ also the $1.5\sigma$ experimentally allowed range of $\sin ^2 \theta _{12}$. Indeed, combining the conditions $0 < \sin ^2 \theta ^e_{12} < 1$ and $|\cos \phi | < 1$, where $\sin ^2 \theta ^e_{12}$ and $\cos \phi$ are given in Eqs. (73) and (74), respectively, and allowing $\sin ^2 \theta _{13}$ to vary in the $3\sigma$ range for NO spectrum, we get restrictions on the value of $\sin ^2 \theta _{12}$, presented in Table 5. We see from the Table that only five out of 18 combinations of the angles $\theta ^{\nu }_{12}$ and $\theta ^{\nu }_{13}$ considered by us satisfy the requirement formulated above. In Table 5 these cases are marked with the subscripts I, II, III, IV, V, while the ones marked with an asterisk contain values of $\sin ^2 \theta _{12}$ allowed at $2\sigma$ [23].

**Table 5:** Ranges of $\sin ^2 \theta _{12}$ obtained from the requirements $(0 < \sin ^2 \theta ^e_{12} < 1) \wedge (-1 < \cos \phi < 1)$ allowing $\sin ^2 \theta _{13}$ to vary in the 3$\sigma$ allowed range for the NO neutrino mass spectrum, quoted in Eq. (5). The cases for which the best fit value of $\sin ^2 \theta _{12} = 0.308$ is within the corresponding allowed ranges are marked with the subscripts I, II, III, IV, V. The cases marked with an asterisk contain values of $\sin ^2 \theta _{12}$ allowed at $2\sigma$ [23]

$\theta ^{\nu }_{12}$	$\theta ^{\nu }_{13} = \pi /20$	$\theta ^{\nu }_{13} = \pi /10$	$\theta ^{\nu }_{13} = \sin ^{-1} (1 / 3)$
$\sin ^{-1} (1 / \sqrt{3})$	$(0.319,0.654)^*$	(0.471, 0.773)	(0.495, 0.789)
$\pi /4$	(0.484, 0.803)	(0.639, 0.897)	(0.662, 0.909)
$-\pi /4$	$(0.197,0.516)_\mathrm{III}$	$(0.103,0.361)_\mathrm{I}$	$(0.091,0.338)_\mathrm{IV}$
$\sin ^{-1} (1 / \sqrt{2 + r})$	$(0.262,0.594)_\mathrm{II}$	(0.409, 0.719)	(0.434, 0.737)
$\sin ^{-1} (\sqrt{3 - r} / 2)$	$(0.331,0.666)^*$	(0.484, 0.784)	(0.508, 0.800)
$\pi /6$	$(0.236,0.564)_\mathrm{V}$	(0.380, 0.692)	(0.404, 0.710)

Equation (67) implies that $\sin ^2 \theta _{23}$ is fixed by the value of $\theta ^{\nu }_{13}$, and for the best fit value of $\sin ^2 \theta _{13}$ and the values of $\theta ^{\nu }_{13} = 0$, $\pi /20$, $\pi /10$, $\sin ^{-1}(1/3)$, considered by us, we get, respectively: $\sin ^2 \theta _{23} = 0.488$, 0.501, 0.537, 0.545. Therefore a measurement of $\sin ^2 \theta _{23}$ with a sufficiently high precision would rule out at least some of the cases with fixed values of $\theta ^{\nu }_{13}$ considered in the literature.

We will perform a statistical analysis of the predictions for $\cos \delta$ in the five cases—I, II, III, IV, V— listed above. The analysis is similar to the one discussed in Sect. 5.1. The only difference is that when we consider the prospective sensitivities on the PMNS mixing angles we will assume $\sin ^2 \theta _{23}$ to have the following potential best fit values: $\sin ^2 \theta _{23} = 0.488$, 0.501, 0.537, 0.545. Note that for the best fit value of $\sin ^2\theta _{13}$, $\sin ^2 \theta _{23} = 0.488$ does not correspond to any of the values of $\theta ^{\nu }_{13}$ in the five cases—I, II, III, IV, V—of interest. Thus, $\sin ^2 \theta _{23} = 0.488$ is not the most probable value in any of the five cases considered: depending on the case, the most probable value is one of the other three values of $\sin ^2 \theta _{23}$ listed above. We include results for $\sin ^2 \theta _{23} = 0.488$ to illustrate how the likelihood function changes when the best fit value of $\sin ^2 \theta _{23}$, determined in a global analysis, differs from the value of $\sin ^2 \theta _{23}$ predicted in a given case.

**Fig. 2:**
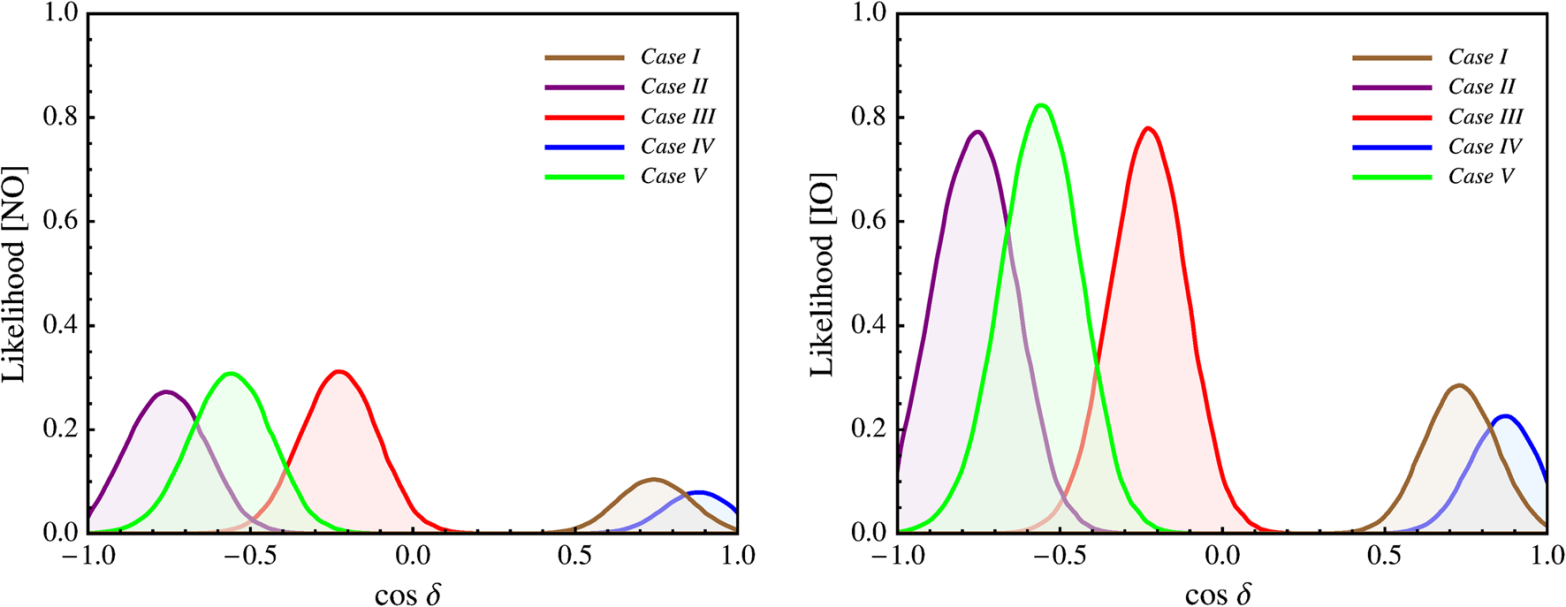
The likelihood function versus $\cos \delta$ for the NO (IO) neutrino mass spectrum in the *left* (*right*) *panel* after marginalising over $\sin ^2\theta _{13}$ for the scheme $\theta ^e_{12} - (\theta ^\nu _{23}, \theta ^\nu _{13}, \theta ^\nu _{12})$ with $[\theta ^\nu _{13}, \theta ^\nu _{12}]$ fixed as $[\pi /10,-\pi /4]$ (Case I), $[\pi /20,b]$ (Case II), $[\pi /20,-\pi /4]$ (Case III), $[a,-\pi /4]$ (Case IV), $[\pi /20,\pi /6]$ (Case V), where $a = \sin ^{-1} (1 / 3)$ and $b = \sin ^{-1} (1 / \sqrt{2 + r})$, *r* being the golden ratio. The figure is obtained using the sum rule in Eq. (77) and the latest results on $\sin ^2\theta _{12}$, $\sin ^2\theta _{13}$, $\sin ^2\theta _{23}$ and $\delta$ from the global analysis of the neutrino oscillation data [23]

In Fig. 2 we show the likelihood function versus $\cos \delta$ for all the cases marked with the subscripts in Table 5. The maxima of the likelihood function in the five cases considered take place at the corresponding values of $\cos \delta$ cited in Table 3. As Fig. 2 clearly indicates, the cases differ not only in the predictions for $\sin ^2 \theta _{23}$, which in the considered set-up is a function of $\sin ^2 \theta ^{\nu }_{13}$ and $\sin ^2 \theta _{13}$, but also in the predictions for $\cos \delta$. Given the values of $\theta _{12}$ and $\theta _{13}$, the positions of the peaks are determined by the values of $\theta ^{\nu }_{12}$ and $\theta ^{\nu }_{13}$

.

The Cases I and IV are disfavoured by the current data because the corresponding values of $\sin ^2\theta _{23} = 0.537$ and 0.545 are disfavoured. The Cases II, III and V are less favoured for the NO neutrino mass spectrum than for the IO spectrum since $\sin ^2\theta _{23} = 0.501$ is less favoured for the first than for the second spectrum.

**Fig. 3:**
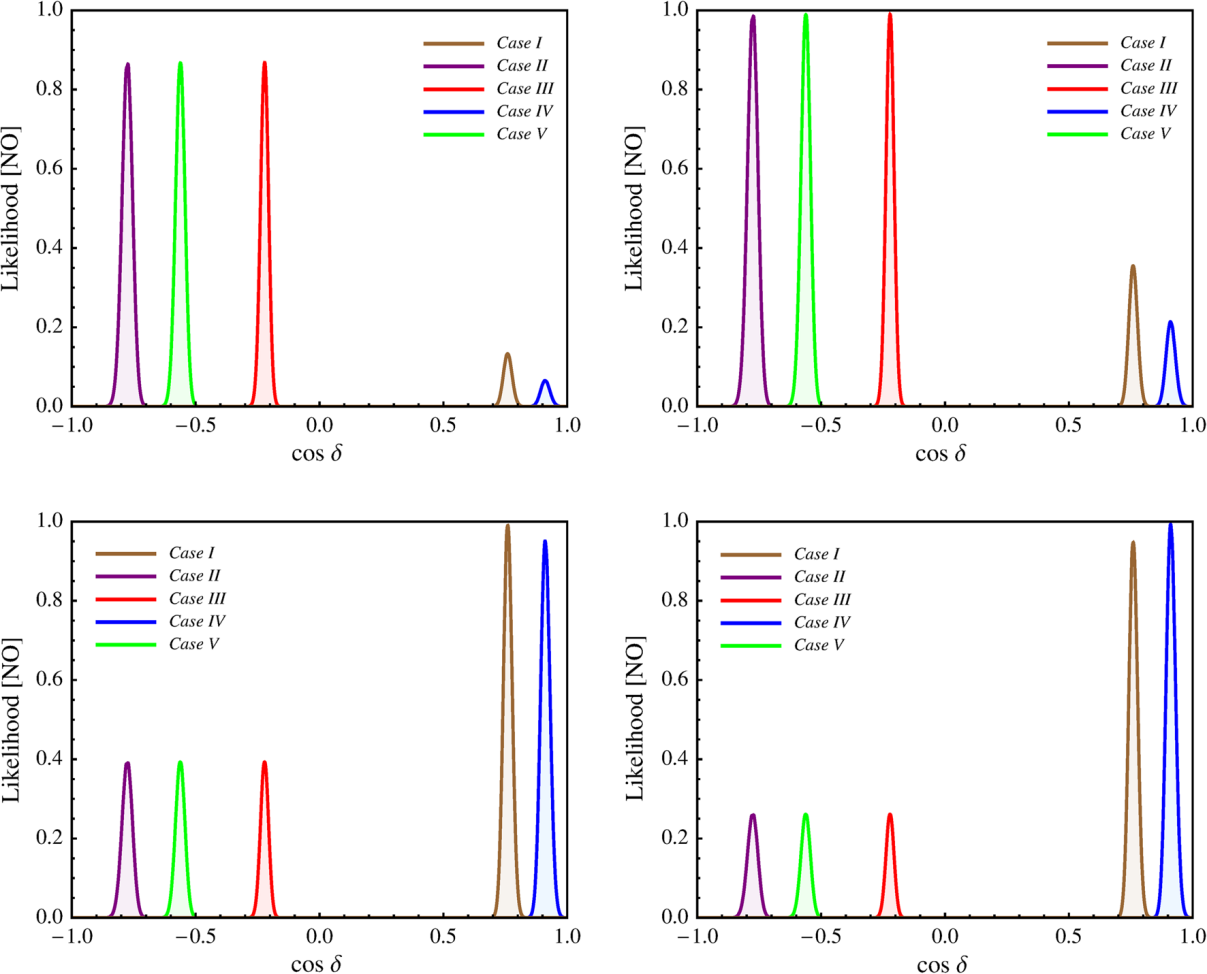
The likelihood function versus $\cos \delta$ for the NO neutrino mass spectrum in the same cases as in Fig. 2, but using the Gaussian approximation with the prospective uncertainties in the measurement of $\sin ^2\theta _{12}$, $\sin ^2\theta _{13}$, $\sin ^2\theta _{23}$, the best fit values for $\sin ^2 \theta _{12}$ and $\sin ^2 \theta _{13}$ as in Eqs. (3) and (5) and the potential best fit values of $\sin ^2 \theta _{23} = 0.488$, 0.501, 0.537, 0.545. *Upper left* (*right*) *panel* $\sin ^2 \theta _{23} = 0.488$ (0.501); *lower left* (*right*) *panel* $\sin ^2 \theta _{23} = 0.537$ (0.545)

In Fig. 3 we show the predictions for $\cos \delta$ using the prospective precision in the measurement of $\sin ^2\theta _{12}$, $\sin ^2\theta _{13}$, $\sin ^2\theta _{23}$, the best fit values for $\sin ^2 \theta _{12}$ and $\sin ^2 \theta _{13}$ as in Eqs. (3) and (5) and the potential best fit values of $\sin ^2 \theta _{23} = 0.488$, 0.501, 0.537, 0.545. The values of $\sin ^2 \theta _{23}$ correspond in the scheme discussed to the best fit value of $\sin ^2 \theta _{13}$ in the cases which are compatible with the current $1.5\sigma$ range of allowed values of $\sin ^2\theta _{12}$. The position of the peaks, obviously, does not depend explicitly on the assumed experimentally determined best fit value of $\sin ^2 \theta _{23}$. For the best fit value of $\sin ^2\theta _{13}$ used, the corresponding sum rule for $\cos \delta$ depends on the given fixed value of $\theta ^{\nu }_{13}$, and via it, on the predicted value of $\sin ^2\theta _{23}$ (see Eqs. (67) and (77)). Therefore, the compatibility of a given case with the considered hypothetical data on $\sin ^2\theta _{23}$ clearly depends on the assumed best fit value of $\sin ^2\theta _{23}$ determined from the data.

As the results shown in Fig. 3 indicate, distinguishing between the Cases I/IV and the other three cases would not require exceedingly high precision measurement of $\cos \delta$. Distinguishing between the Cases II, III and V would be more challenging in terms of the requisite precision on $\cos \delta$. In both cases the precision required will depend, in particular, on the experimentally determined best fit value of $\cos \delta$. As Fig. 3 also indicates, one of the discussed two groups of Cases might be strongly disfavoured by the best fit value of $\sin ^2\theta _{23}$ determined in the future high precision experiments.

**Fig. 4:**
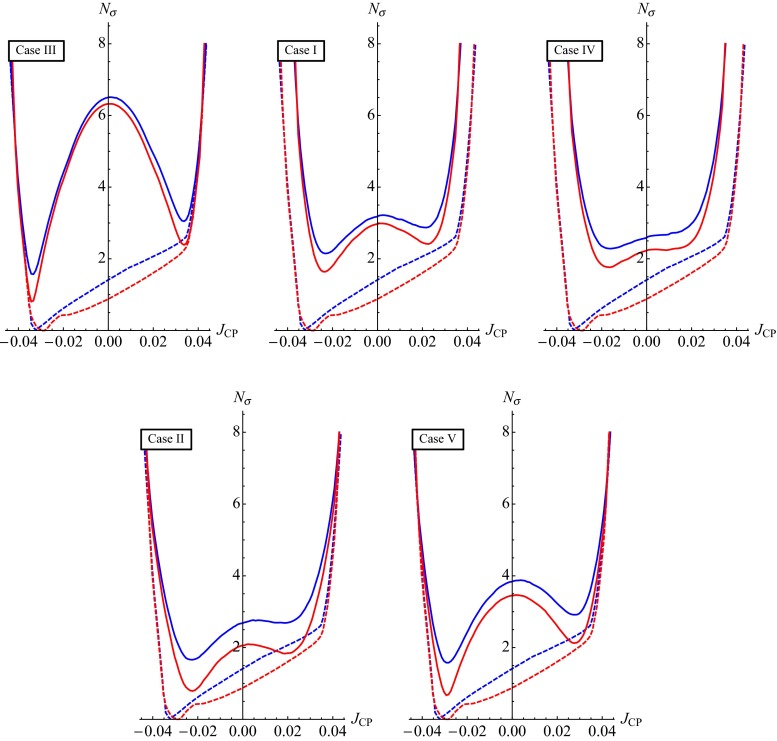
$N_{\sigma } \equiv \sqrt{\chi ^2}$ as a function of $J_\mathrm{CP}$ in the scheme $\theta ^e_{12}-(\theta ^\nu _{23}, \theta ^\nu _{13}, \theta ^\nu _{12})$ with $[\theta ^\nu _{13}, \theta ^\nu _{12}]$ fixed as $[\pi /10,-\pi /4]$ (Case I), $[\pi /20,b]$ (Case II), $[\pi /20,-\pi /4]$ (Case III), $[a,-\pi /4]$ (Case IV), $[\pi /20,\pi /6]$ (Case V), where $a = \sin ^{-1} (1 / 3)$ and $b = \sin ^{-1} (1 / \sqrt{2 + r})$, *r* being the golden ratio. The *dashed lines* represent the results of the global fit [23], while the *solid ones* represent the results we obtain in our set-up. The *blue* (*red*) *lines* are for the NO (IO) neutrino mass spectrum

We have performed also a statistical analysis of the predictions for the rephasing invariant $J_\mathrm{CP}$, minimising $\chi ^2$ for fixed values of $J_\mathrm{CP}$. We give $N_{\sigma } \equiv \sqrt{\chi ^2}$ as a function of $J_\mathrm{CP}$ in Fig. 4. The dashed lines represent the results of the global fit [23], while the solid ones represent the results we obtain for each of the considered cases, minimising the value of $\chi ^2$ in $\theta ^e_{12}$ for a fixed value of $J_\mathrm{CP}$ using Eq. (78). The blue lines correspond to the NO neutrino mass spectrum, while the red ones are for the IO spectrum. The value of $\chi ^2$ in the minimum, which corresponds to the best fit value of $J_\mathrm{CP}$ predicted in the model, allows one to conclude about compatibility of this model with the global neutrino oscillation data. As it can be observed from Fig. 4, the zero value of $J_\mathrm{CP}$ in the Cases III and V is excluded at more than $3\sigma$ with respect to the confidence level of the corresponding minimum. Although in the other three cases the best fit values of $J_\mathrm{CP}$ are relatively large, as their numerical values quoted below show, $J_\mathrm{CP}=0$ is only weakly disfavoured statistically.

The best fit values and the $3\sigma$ ranges of the rephasing invariant $J_\mathrm{CP}$, obtained for the NO neutrino mass spectrum using the current global neutrino oscillation data, in the five cases considered by us are given by

104 $$\begin{aligned}&J_\mathrm{CP}=-0.023, -0.032 \le J_\mathrm{CP}\le 0.029 \quad \mathrm{\, for \, Case \, I}; \end{aligned}$$

105 $$\begin{aligned}&J_\mathrm{CP}=-0.022, -0.035 \le J_\mathrm{CP}\le 0.031 \quad \mathrm{for \, Case \, II}; \end{aligned}$$

106 $$\begin{aligned}&J_\mathrm{CP}=-0.033, -0.039 \le J_\mathrm{CP}\le -0.025,\nonumber \\&\quad 0.030 \le J_\mathrm{CP}\le 0.036 \quad \mathrm{for \, Case \, III}; \end{aligned}$$

107 $$\begin{aligned}&J_\mathrm{CP}=-0.016, -0.028 \le J_\mathrm{CP}\le 0.026 \quad \mathrm{\, for \, Case \, IV}; \end{aligned}$$

108 $$\begin{aligned}&J_\mathrm{CP}=-0.028, -0.037 \le J_\mathrm{CP}\le -0.010,\nonumber \\&\quad 0.018 \le J_\mathrm{CP}\le 0.034\quad \mathrm{for \, Case \, V}. \end{aligned}$$

### The scheme with $\theta ^e_{13}-(\theta ^\nu _{23}, \theta ^\nu _{13}, \theta ^\nu _{12})$ rotations

As in the set-up discussed in Sect. 5.2, we find for the scheme with $\theta ^e_{13}-(\theta ^\nu _{23}, \theta ^\nu _{13}, \theta ^\nu _{12})$ rotations that only particular values of $\theta ^{\nu }_{12}$ and $\theta ^{\nu }_{13}$ allow one to obtain the current best fit value of $\sin ^2 \theta _{12}$. Combining the requirements $0 < \sin ^2 \theta ^e_{13} < 1$ and $|\cos \omega | < 1$, where $\sin ^2 \theta ^e_{13}$ and $\cos \omega$ are given in Eqs. (89) and (90), respectively, and allowing $\sin ^2 \theta _{13}$ to vary in its $3\sigma$ allowed range corresponding to the NO spectrum, we get restrictions on the value of $\sin ^2 \theta _{12}$, presented in Table 6. It follows from the results in Table 6 that only for five out of 18 combinations of the angles $\theta ^{\nu }_{12}$ and $\theta ^{\nu }_{13}$, the best fit value of $\sin ^2 \theta _{12} = 0.308$ and the $1.5\sigma$ experimentally allowed interval of values of $\sin ^2 \theta _{12}$ are inside the allowed ranges. In Table 6 these cases are marked with the subscripts I–V, while in the case marked with an asterisk, the allowed range contains values of $\sin ^2 \theta _{12}$ allowed at $2\sigma$ [23].

**Table 6:** Ranges of $\sin ^2 \theta _{12}$ obtained from the requirements $(0 < \sin ^2 \theta ^e_{13} < 1) \wedge (-1 < \cos \omega <1)$ allowing $\sin ^2 \theta _{13}$ to vary in the 3$\sigma$ allowed range for the NO neutrino mass spectrum, quoted in Eq. (5). The cases for which the best fit value of $\sin ^2 \theta _{12} = 0.308$ is within the corresponding allowed ranges are marked with the subscripts I, II, III, IV, V. The case marked with an asterisk contains values of $\sin ^2 \theta _{12}$ allowed at $2\sigma$ [23]

$\theta ^{\nu }_{12}$	$\theta ^{\nu }_{13} = \pi /20$	$\theta ^{\nu }_{13} = \pi /10$	$\theta ^{\nu }_{13} = \sin ^{-1} (1 / 3)$
$\sin ^{-1} (1 / \sqrt{3})$	$(0.081,0.348)_\mathrm{III}$	(0.024, 0.209)	(0.019, 0.189)
$\pi /4$	$(0.197,0.516)_\mathrm{I}$	$(0.103,0.361)_\mathrm{IV}$	$(0.091,0.338)_\mathrm{II}$
$-\pi /4$	(0.484, 0.803)	(0.639, 0.897)	(0.662, 0.909)
$\sin ^{-1} (1 / \sqrt{2 + r})$	$(0.051,0.291)^*$	(0.009, 0.161)	(0.006, 0.143)
$\sin ^{-1} (\sqrt{3 - r} / 2)$	$(0.089,0.361)_\mathrm{V}$	(0.028, 0.220)	(0.022, 0.200)
$\pi /6$	(0.038, 0.264)	(0.004, 0.140)	(0.002, 0.123)

The values of $\sin ^2 \theta _{23}$ in this model depend on the reactor angle $\theta _{13}$ and $\theta ^{\nu }_{13}$ through Eq. (83). Using the best fit value of $\sin ^2 \theta _{13}$ for the NO spectrum and Eq. (83), we find $\sin ^2 \theta _{23} = 0.512$, 0.499, 0.463, 0.455 for $\theta ^{\nu }_{13} = 0$, $\pi /20$, $\pi /10$, $\sin ^{-1} (1 / 3)$, respectively. Thus, in the scheme under discussion $\sin ^2 \theta _{23}$ decreases with the increase of $\theta ^{\nu }_{13}$, which is in contrast to the behaviour of $\sin ^2 \theta _{23}$ in the set-up discussed in the preceding subsection. As we have already remarked, a measurement of $\sin ^2 \theta _{23}$ with a sufficiently high precision, or at least the determination of the octant of $\theta _{23}$, would allow one to exclude some of the values of $\theta ^{\nu }_{13}$ considered in the literature.

**Fig. 5:**
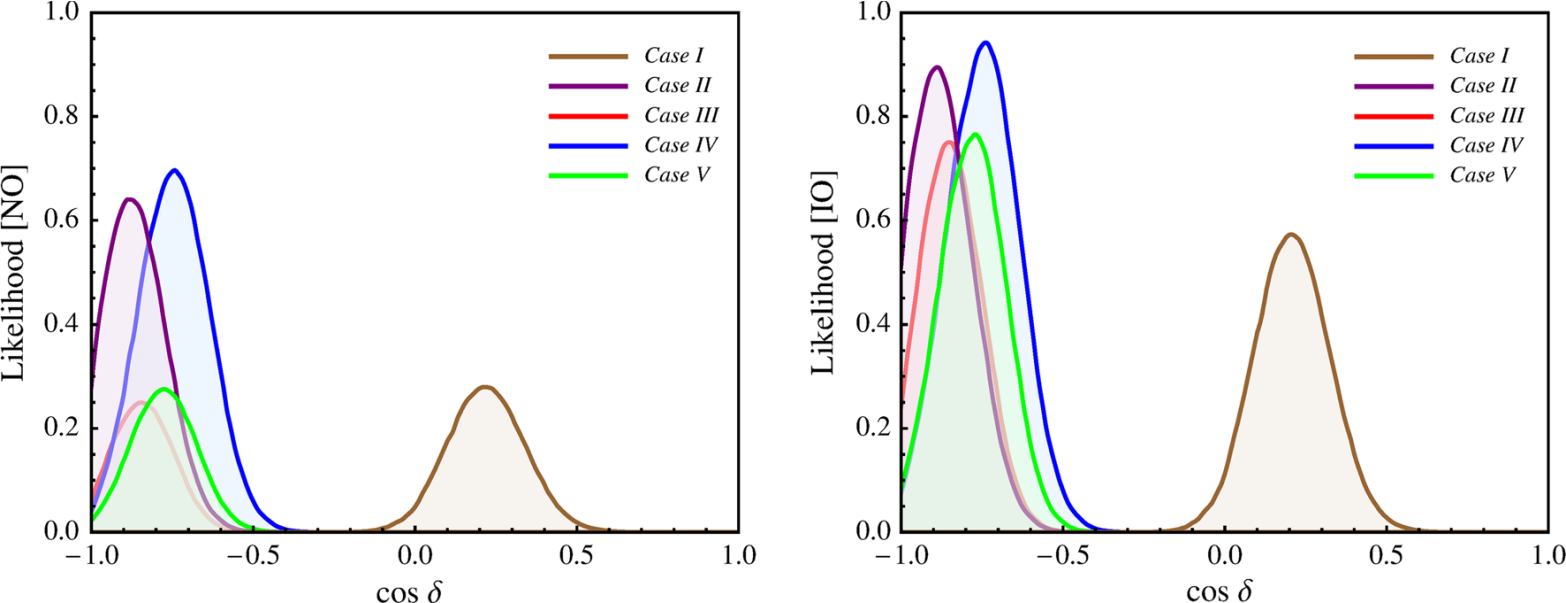
The likelihood function versus $\cos \delta$ for the NO (IO) neutrino mass spectrum in the *left* (*right*) *panel* after marginalising over $\sin ^2\theta _{13}$ for the scheme $\theta ^e_{13} - (\theta ^\nu _{23}, \theta ^\nu _{13}, \theta ^\nu _{12})$ with $[\theta ^\nu _{13}, \theta ^\nu _{12}]$ fixed as $[\pi /20,\pi /4]$ (Case I), $[a,\pi /4]$ (Case II), $[\pi /20,c]$ (Case III), $[\pi /10,\pi /4]$ (Case IV), $[\pi /20,d]$ (Case V). We have defined $a = \sin ^{-1} (1 / 3)$, $c = \sin ^{-1} (1 / \sqrt{3})$ and $d = \sin ^{-1} (\sqrt{3 - r}/2)$, *r* being the golden ratio. The figure is obtained using the sum rule in Eq. (93) and the latest results on $\sin ^2\theta _{12}$, $\sin ^2\theta _{13}$, $\sin ^2\theta _{23}$ and $\delta$ from the global analysis of the neutrino oscillation data [23]

**Fig. 6:**
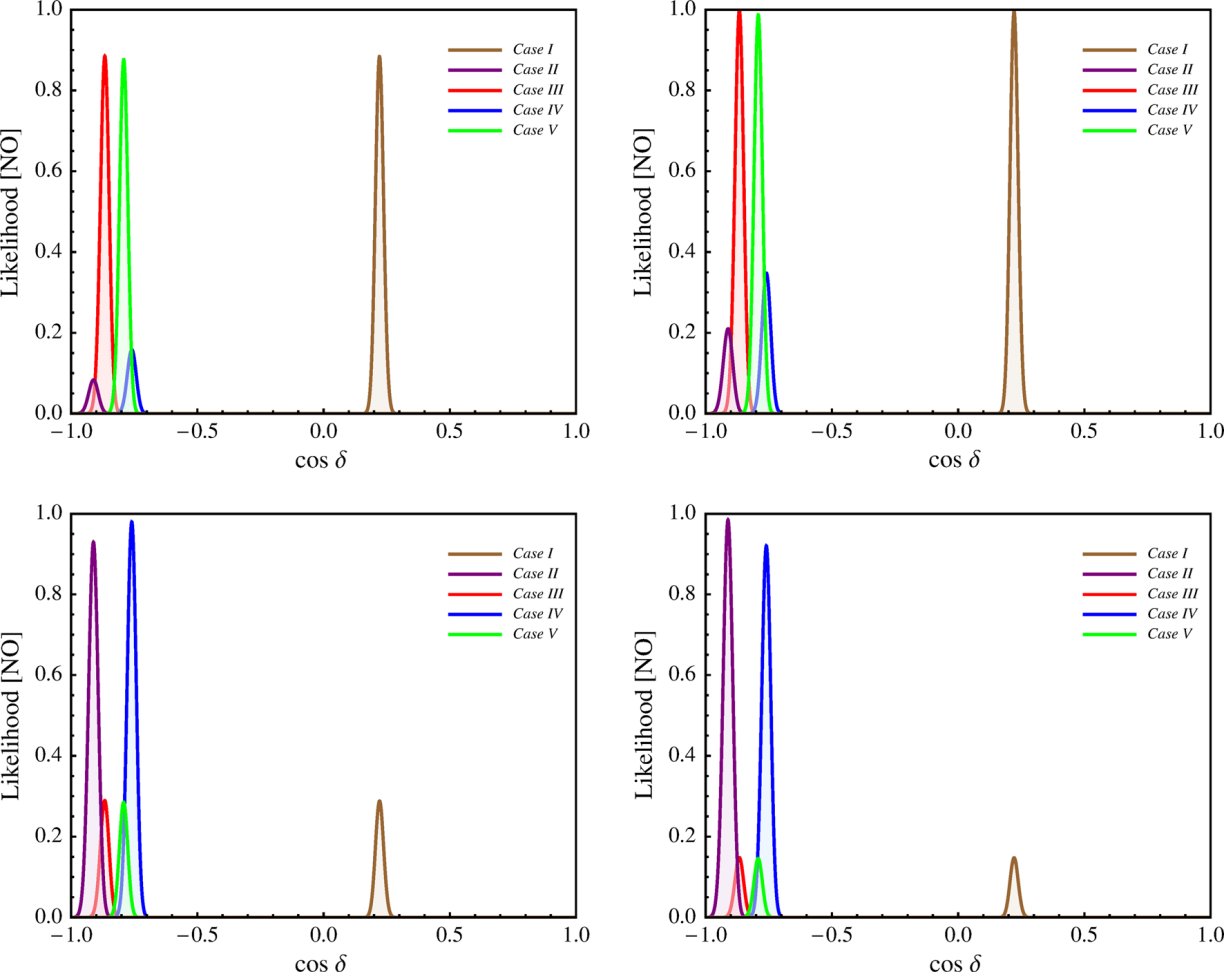
The likelihood function versus $\cos \delta$ for the NO neutrino mass spectrum in the cases described in Fig. 5, but within the Gaussian approximation. The *upper left* (*right*) *panel* corresponds to the potential best fit value of $\sin ^2 \theta _{23} = 0.512$ (0.499), while the *lower left* (*right*) *panel* is obtained for the potential best fit value of $\sin ^2\theta _{23} = 0.463$ (0.455); the best fit values of $\sin ^2\theta _{12}$ and $\sin ^2\theta _{13}$ correspond to those quoted in Eqs. (3) and (5). The figure is obtained using the prospective uncertainties in the values of $\sin ^2 \theta _{12}$, $\sin ^2 \theta _{13}$ and $\sin ^2 \theta _{23}$

The statistical analyses for $\delta$ and $J_\mathrm{CP}$ performed in the present subsection are similar to those performed in the previous subsections. In particular, we show in Fig. 5 the dependence of the likelihood function on $\cos \delta$ using the current knowledge on the PMNS mixing angles and the Dirac CPV phase from the latest global fit results. Due to the very narrow prediction for $\sin ^2 \theta _{23}$ in this set-up, the prospective sensitivity likelihood curve depends strongly on the assumed best fit value of $\sin ^2 \theta _{23}$. For this reason we present in Fig. 6 the predictions for $\cos \delta$ using the prospective sensitivities on the mixing angles, the best fit values for $\sin ^2 \theta _{12}$ and $\sin ^2 \theta _{13}$ as in Eqs. (3) and (5) and the potential best fit values of $\sin ^2 \theta _{23} = 0.512$, 0.499, 0.463, 0.455. We use the value of $\sin ^2 \theta _{23} = 0.512$, corresponding to $\theta ^{\nu }_{13} = 0$, for the same reason we used the value of $\sin ^2 \theta _{23} = 0.488$ in the analysis in the preceding subsection, where we gave also a detailed explanation.

As Fig. 6 clearly shows, the position of the peaks does not depend on the assumed best fit value of $\sin ^2 \theta _{23}$. However, the height of the peaks reflects to what degree the model is disfavoured due to the difference between the assumed best fit value of $\sin ^2 \theta _{23}$ and the value predicted in the corresponding set-up.

**Fig. 7:**
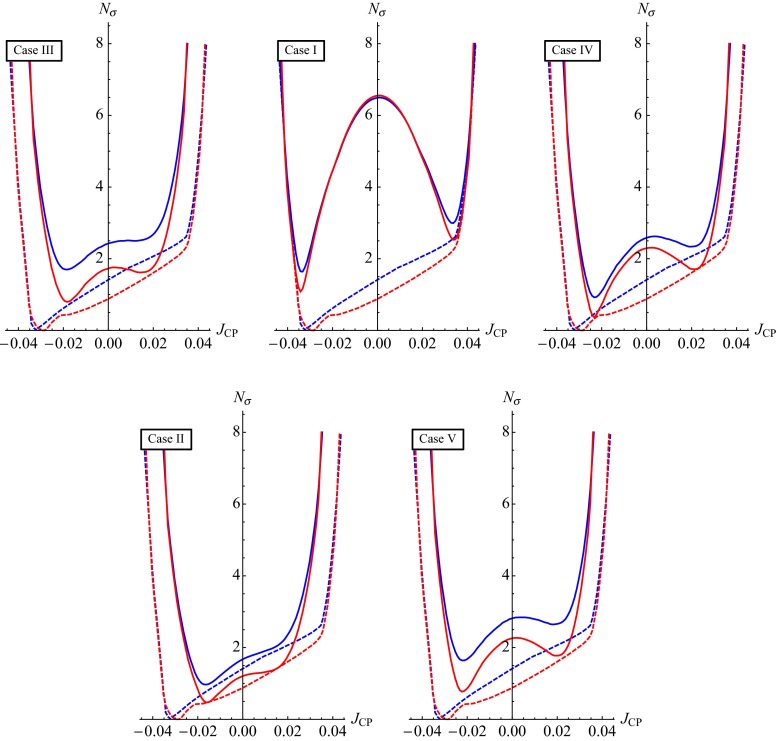
The same as in Fig. 4, but for the scheme $\theta ^e_{13}-(\theta ^\nu _{23}, \theta ^\nu _{13}, \theta ^\nu _{12})$ with $[\theta ^\nu _{13}, \theta ^\nu _{12}]$ given by $[\pi /20,\pi /4]$ (Case I), $[a,\pi /4]$ (Case II), $[\pi /20,c]$ (Case III), $[\pi /10,\pi /4]$ (Case IV), $[\pi /20,d]$ (Case V), where $a = \sin ^{-1} (1 / 3)$, $c = \sin ^{-1} (1 / \sqrt{3})$ and $d = \sin ^{-1} (\sqrt{3 - r}/2)$, *r* being the golden ratio. The *dashed lines* represent the results of the global fit [23], while the *solid ones* represent the results we obtain in our set-up. The *blue* (*red*) *lines* are for the NO (IO) neutrino mass spectrum

The results shown in Fig. 6 clearly indicate that (i) the measurement of $\cos \delta$ can allow one to distinguish between the Case I and the other four cases; (ii) distinguishing between the Cases II/III and the Cases IV/V might be possible, but is very challenging in terms of the precision on $\cos \delta$ required to achieve that; and (iii) distinguishing between the Cases II and III (the Cases IV and V) seems practically impossible. Some of, or even all, these cases would be strongly disfavoured if the best fit value of $\sin ^2\theta _{23}$ determined with the assumed high precision in the future experiments were relatively large, say, $\sin ^2\theta _{23} \gtrsim 0.54$.

The results on the predictions for the rephasing invariant $J_\mathrm{CP}$ are presented in Fig. 7, where we show the dependence of $N_{\sigma } \equiv \sqrt{\chi ^2}$ on $J_\mathrm{CP}$. It follows from the results presented in Fig. 7, in particular, that $J_\mathrm{CP} =0$ is excluded at more than $3\sigma$ with respect to the confidence level of the corresponding minimum only in the Case I. For the rephasing invariant $J_\mathrm{CP}$, using the current global neutrino oscillation data, we find for the different cases considered the following best fit values and $3\sigma$ ranges for the NO neutrino mass spectrum:

109 $$\begin{aligned}&J_\mathrm{CP}=-0.033, -0.039 \le J_\mathrm{CP}\le -0.025,\nonumber \\&\quad 0.029 \le J_\mathrm{CP}\le 0.037 \quad \mathrm{for \, Case \, I}; \end{aligned}$$

110 $$\begin{aligned}&J_\mathrm{CP}=-0.016, -0.028 \le J_\mathrm{CP}\le 0.025 \quad \mathrm{for \, Case \, II}; \end{aligned}$$

111 $$\begin{aligned}&J_\mathrm{CP}=-0.018, -0.029 \le J_\mathrm{CP}\le 0.026 \quad \mathrm{for \, Case \, III}; \end{aligned}$$

112 $$\begin{aligned}&J_\mathrm{CP}=-0.023, -0.031 \le J_\mathrm{CP}\le 0.029 \quad \mathrm{for \, Case \, IV}; \end{aligned}$$

113 $$\begin{aligned}&J_\mathrm{CP}=-0.022, -0.030 \le J_\mathrm{CP}\le 0.028 \quad \mathrm{for \, Case \, V}. \end{aligned}$$

## Summary and conclusions

In the present article we have derived predictions for the Dirac phase $\delta$ present in the $3\times 3$ unitary neutrino mixing matrix $U = U_e^{\dagger } \, U_{\nu } = (\tilde{U}_{e})^\dagger \, \Psi \tilde{U}_{\nu } \, Q_0$, where $U_e$ ($\tilde{U}_e$) and $U_{\nu }$ ($\tilde{U}_\nu$) are $3\times 3$ unitary (CKM-like) matrices which arise from the diagonalisation, respectively, of the charged lepton and the neutrino mass matrices, and $\Psi$ and $Q_0$ are diagonal phase matrices each containing in the general case two physical CPV phases. The phases in the matrix $Q_0$ contribute to the Majorana phases in the PMNS matrix. After performing a systematic search, we have considered forms of $\tilde{U}_e$ and $\tilde{U}_{\nu }$ allowing us to express $\delta$ as a function of the PMNS mixing angles, $\theta _{12}$, $\theta _{13}$ and $\theta _{23}$, present in *U*, and the angles contained in $\tilde{U}_{\nu }$. We have derived such sum rules for $\cos \delta$ in the cases of forms for which the sum rules of interest do not exist in the literature. More specifically, we have derived new sum rules for $\cos \delta$ in the following cases:

(i) $$U \!=\! R_{12}(\theta ^e_{12})\Psi R_{23}(\theta ^{\nu }_{23}) R_{12}(\theta ^{\nu }_{12}) Q_0$$ ($$\theta ^e_{12} - (\theta ^\nu _{23}, \theta ^\nu _{12})$$ scheme),
(ii) $$U \!=\! R_{13}(\theta ^e_{13})\Psi R_{23}(\theta ^{\nu }_{23}) R_{12}(\theta ^{\nu }_{12}) Q_0$$ ($$\theta ^e_{13} - (\theta ^\nu _{23}, \theta ^\nu _{12})$$ scheme),
(iii) $$U =R_{13}(\theta ^e_{13})R_{23}(\theta ^e_{23})\Psi R_{23}(\theta ^{\nu }_{23}) R_{12}(\theta ^{\nu }_{12}) Q_0$$ ($$(\theta ^e_{13},\theta ^e_{23}) - (\theta ^\nu _{23}, \theta ^\nu _{12})$$ scheme),
(iv) $$U =R_{12}(\theta ^e_{12}) R_{13}(\theta ^e_{13}) \Psi R_{23}(\theta ^{\nu }_{23}) R_{12}(\theta ^{\nu }_{12}) Q_0$$ ($$(\theta ^e_{12},\theta ^e_{13}) - (\theta ^\nu _{23}, \theta ^\nu _{12})$$ scheme),
(v) $$U =R_{12}(\theta ^e_{12})\Psi R_{23}(\theta ^{\nu }_{23}) R_{13}(\theta ^{\nu }_{13}) R_{12}(\theta ^{\nu }_{12}) Q_0$$ ($$\theta ^e_{12} - (\theta ^\nu _{23}, \theta ^\nu _{13}, \theta ^\nu _{12})$$ scheme), and
(vi) $$U = R_{13}(\theta ^e_{13}) \Psi R_{23}(\theta ^{\nu }_{23}) R_{13}(\theta ^{\nu }_{13}) R_{12}(\theta ^{\nu }_{12}) Q_0$$ ($$\theta ^e_{13} - (\theta ^\nu _{23}, \theta ^\nu _{13}, \theta ^\nu _{12})$$ scheme),

where $R_{ij}$ are real orthogonal matrices describing rotations in the *i*–*j* plane, and $\theta ^e_{ij}$ and $\theta ^{\nu }_{ij}$ stand for the rotation angles contained in $\tilde{U}_e$ and $\tilde{U}_{\nu }$, respectively. In the sum rules $\cos \delta$ is expressed, in general, in terms of the three angles of the PMNS matrix, $\theta _{12}$, $\theta _{13}$ and $\theta _{23}$, measured, e.g., in the neutrino oscillation experiments, and the angles in $\tilde{U}_{\nu }$, which are assumed to have fixed known values. In the case of the scheme (iv), $\cos \delta$ depends in addition on an a priori unknown phase $\omega$, whose value can only be fixed in a self-consistent model of neutrino mass generation. A summary of the sum rules derived in the present article is given in Table 1.

To obtain predictions for $\cos \delta$, $\delta$ and the $J_\mathrm{CP}$ factor, which controls the magnitude of the CP-violating effects in neutrino oscillations, we have considered several forms of $\tilde{U}_{\nu }$ determined by, or associated with, symmetries, for which the angles in $\tilde{U}_{\nu }$ have specific values. More concretely, in the cases (i)–(iv), we have performed analyses for the TBM, BM (LC), GRA, GRB, and HG forms of $\tilde{U}_{\nu }$. For all these forms we have $\theta ^{\nu }_{23} = -\pi /4$ and $\theta ^{\nu }_{13} = 0$. The forms differ by the value of the angle $\theta ^{\nu }_{12}$, which for the different forms of interest was given in Sect. 1. In the schemes (v) and (vi) with non-zero fixed values of $\theta ^{\nu }_{13}$, which are also inspired by certain types of flavour symmetries, we have considered three representative values of $\theta ^{\nu }_{13}$ discussed in the literature, $\theta ^{\nu }_{13} = \pi /20,~\pi /10$ and $a = \sin ^{-1} (1 / 3)$, in combination with specific values of $\theta ^{\nu }_{12}$ – altogether five sets of different pairs of values of $[\theta ^{\nu }_{13},\theta ^{\nu }_{12}]$ in each of the two schemes. They are given in Table 3.

We first obtained predictions for $\cos \delta$ and $\delta$ using the current best fit values of $\sin ^2\theta _{12}$, $\sin ^2\theta _{13}$ and $\sin ^2\theta _{23}$, given in Eqs. (3)–(5). They are summarised in Tables 3 and 4. The quoted values of $\cos \delta$ and $\delta$ for the scheme (iv) are for $\omega = 0$. For completeness, in Tables 3 and 4 we have presented results also for

(vii) the $\theta ^e_{12}-(\theta ^\nu _{23}, \theta ^\nu _{12})$ scheme (in which $(\tilde{U}_{e})^\dagger = R_{12}(\theta ^e_{12})$, $\tilde{U}_{\nu }= R_{23}(\theta ^{\nu }_{23}) R_{12}(\theta ^{\nu }_{12})$), and
(viii) the $(\theta ^e_{12},\theta ^e_{23})-(\theta ^\nu _{23}, \theta ^\nu _{12})$ scheme (in which $(\tilde{U}_{e})^\dagger = R_{12}(\theta ^e_{12})R_{23}(\theta ^e_{23})$, $\tilde{U}_{\nu }= R_{23}(\theta ^{\nu }_{23}) R_{12}(\theta ^{\nu }_{12})$).

For these two schemes results were given earlier in [45]. We have updated the predictions obtained in [45] using the best fit values of $\sin ^2\theta _{12}$, $\sin ^2\theta _{13}$ and $\sin ^2\theta _{23}$, found in the most recent analyses of the neutrino oscillation data.

We have not presented predictions for the BM (LC) symmetry form of $\tilde{U}_{\nu }$ in Tables 3 and 4, because for the current best fit values of $\sin ^2 \theta _{12}$, $\sin ^2 \theta _{23}$, $\sin ^2 \theta _{13}$ the corresponding sum rules were found to give unphysical values of $\cos \delta$ (see, however, Refs. [49, 50]).

We have found that the predictions for $\cos \delta$ of the $\theta ^e_{12}-(\theta ^\nu _{23}, \theta ^\nu _{12})$ and $\theta ^e_{13}-(\theta ^\nu _{23}, \theta ^\nu _{12})$ schemes for each of the symmetry forms of $\tilde{U}_{\nu }$ considered differ only by sign. The $\theta ^e_{12}-(\theta ^\nu _{23}, \theta ^\nu _{12})$ scheme and the $(\theta ^e_{12},\theta ^e_{13}) - (\theta ^\nu _{23}, \theta ^\nu _{12})$ scheme with $\omega = 0$ provide very similar predictions for $\cos \delta$.

In the schemes with three rotations in $\tilde{U}_{\nu }$ we consider, $\cos \delta$ is predicted to have values which typically differ significantly (being larger in absolute value) from the values predicted by the schemes with two rotations in $\tilde{U}_{\nu }$ discussed by us, the only exceptions being two cases (see Table 3).

We have found also that the predictions for $\cos \delta$ of the set-ups denoted as $(\theta ^e_{12},\theta ^e_{23}) - (\theta ^\nu _{23}, \theta ^\nu _{12})$ and $(\theta ^e_{13},\theta ^e_{23})-(\theta ^\nu _{23}, \theta ^\nu _{12})$ differ for each of the symmetry forms of $\tilde{U}_{\nu }$ considered both by sign and magnitude. If the best fit value of $\theta _{23}$ were $\pi /4$, these predictions would differ only by sign. In the case of the $(\theta ^e_{12},\theta ^e_{13}) - (\theta ^\nu _{23}, \theta ^\nu _{12})$ scheme, the predictions for $\cos \delta$ depend on the value chosen of the phase $\omega$.

We have performed next a statistical analysis of the predictions (a) for $\cos \delta$ and $J_\mathrm{CP}$ using the latest results of the global fit analysis of neutrino oscillation data, and (b) for $\cos \delta$ using prospective sensitivities on the PMNS mixing angles. This was done by constructing likelihood functions in the two cases.

For the reasons related to the dependence of $\cos \delta$ on $\omega$ we did not present results of the statistical analysis for the $(\theta ^e_{12},\theta ^e_{13})-(\theta ^\nu _{23}, \theta ^\nu _{12})$ scheme. This can be done in self-consistent models of neutrino mixing, in which the value of the phase $\omega$ is fixed by the model.

We have found also that in the case of the $\theta ^e_{12} - (\theta ^\nu _{23}, \theta ^\nu _{12})$ scheme, the results for $\chi ^2$ as a function of $\delta$ or $J_\mathrm{CP}$ are rather similar to those obtained in [49, 50] in the $(\theta ^e_{12},\theta ^e_{23})$–$(\theta ^\nu _{23}, \theta ^\nu _{12})$ set-up. The main difference between these two schemes is the predictions for $\sin ^2 \theta _{23}$, which can deviate only by approximately $0.5 \sin ^2 \theta _{13}$ from 0.5 in the first scheme, and by a significantly larger amount in the second. Similar conclusions hold comparing the results for the $\theta ^e_{13} - (\theta ^\nu _{23},\theta ^\nu _{12})$ scheme and in the $(\theta ^e_{13},\theta ^e_{23})-(\theta ^\nu _{23},\theta ^\nu _{12})$ scheme. Therefore, in what concerns these four schemes, given the above conclusions and the fact that for the $(\theta ^e_{12},\theta ^e_{23})$–$(\theta ^\nu _{23},\theta ^\nu _{12})$ scheme detailed results already exist in the literature (see [49, 50]), we have presented results of statistical analysis of the predictions for $\cos \delta$ and the $J_\mathrm{CP}$ factor only for the $(\theta ^e_{13},\theta ^e_{23}) - (\theta ^\nu _{23},\theta ^\nu _{12})$ scheme. This was done for the five symmetry forms considered – TBM, BM (LC), GRA, GRB and HG. We have found, in particular, that for a given symmetry form, $\cos \delta$ is predicted to have opposite sign to that predicted in the $(\theta ^e_{12},\theta ^e_{23})-(\theta ^\nu _{23},\theta ^\nu _{12})$ scheme. Thus, in the $(\theta ^e_{13},\theta ^e_{23}) - (\theta ^\nu _{23}, \theta ^\nu _{12})$ scheme analysed in the present article, one has $\cos \delta > 0$ in the TBM, GRB and BM (LC) cases, and $\cos \delta <0$ in the cases of GRA and HG symmetry forms of $\tilde{U}_{\nu }$. As in the $(\theta ^e_{12},\theta ^e_{23}) - (\theta ^\nu _{23}, \theta ^\nu _{12})$ set-up, there are significant overlaps between the predictions for $\cos \delta$ for the TBM and GRB forms, and for the GRA and HG forms, respectively. The BM (LC) case is disfavoured at more than $2\sigma$ confidence level. Due to the fact that the best fit value of $\sin ^2\theta _{23} < 0.5$, the predictions for $\cos \delta$ for each symmetry form, obtained in the discussed two set-ups differ not only by sign but also in absolute value. We found also that in the $(\theta ^e_{13},\theta ^e_{23}) - (\theta ^\nu _{23}, \theta ^\nu _{12})$ scheme relatively large CP-violating effects in neutrino oscillations are predicted for all symmetry forms considered, the only exception being the case of the BM symmetry form.

In the case of the $\theta ^e_{12}-(\theta ^\nu _{23}, \theta ^\nu _{13}, \theta ^\nu _{12})$ and $\theta ^e_{13} - (\theta ^\nu _{23}, \theta ^\nu _{13}, \theta ^\nu _{12})$ schemes we have performed statistical analyses of the predictions for $\cos \delta$ and the $J_\mathrm{CP}$ factor for the five sets of values of the angles $[\theta ^\nu _{13}, \theta ^\nu _{12}]$ listed in Tables 3 and 4. These sets differ for the two schemes. For the values of $[\theta ^\nu _{13},\theta ^\nu _{12}]$ given in Tables 3 and 4, the allowed intervals of values of $\sin ^2\theta _{12}$ in the two schemes, in particular, satisfy the requirement that they contain the best fit value and the $1.5\sigma$ experimentally allowed range of $\sin ^2\theta _{12}$. In the discussed two schemes the value of $\sin ^2\theta _{23}$ is determined by the values of $\theta _{13}$, $\theta ^\nu _{13}$ and $\theta ^\nu _{23}$ (see Table 2). In the statistical analyses we have performed $\theta ^\nu _{23}$ was set to $(-\pi /4)$. Setting $\sin ^2\theta _{13}$ to its best fit value, in the scheme $\theta ^e_{12}-(\theta ^\nu _{23}, \theta ^\nu _{13}, \theta ^\nu _{12})$ and for $\theta ^{\nu }_{13} = 0$, $\pi /20$, $\pi /10$ and $\sin ^{-1}(1/3)$ we found, respectively: $\sin ^2 \theta _{23} = 0.488$, 0.501, 0.537 and 0.545. For the same values of $\sin ^2\theta _{13}$ and $\theta ^{\nu }_{13}$ we obtained in the scheme $\theta ^e_{13}-(\theta ^\nu _{23}, \theta ^\nu _{13}, \theta ^\nu _{12})$: $\sin ^2 \theta _{23} = 0.512$, 0.499, 0.463, 0.455.

Further, the statistical analyses we have performed showed that for each of the two schemes, the five cases considered form two groups for which $\cos \delta$ differs in sign and in magnitude (Figs. 2, 5). This suggests that distinguishing between the two groups for each of the two schemes considered could be achieved with a not very demanding (in terms of precision) measurement of $\cos \delta$. In the analyses performed using the prospective sensitivities on $\sin ^2\theta _{12}$, $\sin ^2\theta _{13}$ and $\sin ^2\theta _{23}$, assuming the current best fit values of $\sin ^2\theta _{12}$, $\sin ^2\theta _{13}$ will not change, we have chosen as potential best fit values of $\sin ^2 \theta _{23}$ those predicted by the two schemes in the five cases considered (the values are listed in the preceding paragraph). These analyses have revealed, in particular, that for each of the two schemes, distinguishing between the cases inside the two groups which provide opposite sign predictions for $\cos \delta$ would be more challenging in terms of the requisite precision on $\cos \delta$; for certain pairs of cases predicting $\cos \delta < -0.5$ in the scheme $\theta ^e_{13} - (\theta ^\nu _{23}, \theta ^\nu _{13}, \theta ^\nu _{12})$, this seems impossible to achieve in practice. These conclusions are well illustrated by Figs. 3 and 6. However, we have found that, depending on the chosen potential best fit value of $\sin ^2 \theta _{23}$, some of the cases are strongly disfavoured. Thus, a high precision measurement of $\sin ^2 \theta _{23}$ would certainly rule out some of (if not all) the cases of the two schemes we have considered.

The analysis performed of the predictions for the $J_\mathrm{CP}$ factor showed that in the $\theta ^e_{12}-(\theta ^\nu _{23}, \theta ^\nu _{13}, \theta ^\nu _{12})$ set-up, the CP-conserving value of $J_\mathrm{CP} = 0$ is excluded at more than $3\sigma$ with respect to the confidence level of the corresponding minimum, in two cases, namely, for $[\theta ^{\nu }_{13}, \theta ^{\nu }_{12}] = [\pi /20, - \pi /4]$, $[\pi /20,\pi /6]$ (denoted in the text as Cases III and V). In the other three cases in spite of the relatively large predicted best fit values of $J_\mathrm{CP}$, $J_\mathrm{CP} = 0$ is only weakly disfavored (Fig. 4). For the $\theta ^e_{13} - (\theta ^\nu _{23}, \theta ^\nu _{13}, \theta ^\nu _{12})$ scheme, $J_\mathrm{CP} = 0$ is excluded at more than $3\sigma$ (with respect to the confidence level of the corresponding minimum), only in one case (denoted as Case I in the text), namely, for $[\theta ^{\nu }_{13}, \theta ^{\nu }_{12}] = [\pi /20, \pi /4]$ (Fig. 7).

The results obtained in the present article confirm the conclusion reached in earlier similar studies that the measurement of the Dirac phase in the PMNS mixing matrix, together with an improvement of the precision on the mixing angles $\theta _{12}$, $\theta _{13}$ and $\theta _{23}$, can provide unique information as regards the possible existence of symmetry in the lepton sector. These measurements could also provide an indication about the structure of the matrix $\tilde{U}_e$ originating from the charged lepton sector, and thus about the charged lepton mass matrix.

## Figures and Tables

**Fig. 8:**
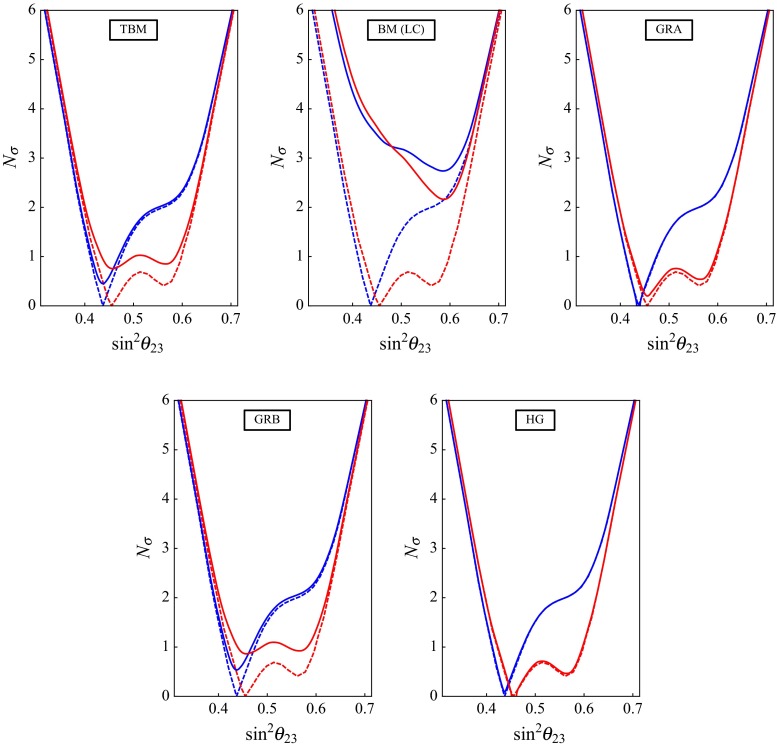
$N_{\sigma } \equiv \sqrt{\chi ^2}$ as a function of $\sin ^2 \theta _{23}$ in the scheme $(\theta ^e_{13},\theta ^e_{23}) - (\theta ^\nu _{23}, \theta ^\nu _{12})$. The *dashed lines* represent the results of the global fit [23], while the *solid ones* represent the results we obtain for the TBM, BM (LC), GRA (*upper left*, *central*, *right panels*), GRB and HG (*lower left* and *right panels*) neutrino mixing symmetry forms. The *blue* (*red*) *lines* are for the NO (IO) neutrino mass spectrum

## Appendix A: Statistical details

In order to perform a statistical analysis of the schemes considered we use as input the latest results on $\sin ^2\theta _{12}$, $\sin ^2\theta _{13}$, $\sin ^2\theta _{23}$ and $\delta$, obtained in the global analysis of the neutrino oscillation data performed in [23]. The aim is to derive the allowed ranges for $\cos \delta$ and $J_\mathrm{CP}$, predicted on the basis of the current data on the neutrino mixing parameters for each scheme considered. For this purpose we construct the $\chi ^2$ function in the following way: $\chi ^2(\{x_i\}) = \sum _i \chi _i^2(x_i)$, with $x_i = \{\sin ^2 \theta _{12},\sin ^2 \theta _{13},\sin ^2 \theta _{23},\delta \}$. The functions $\chi ^2_i$ have been extracted from the 1-dimensional projections given in [23] and, thus, the correlations between the oscillation parameters have been neglected. This approximation is sufficiently precise since it allows one to reproduce the contours in the planes $(\sin ^2 \theta _{23} , \delta )$, $(\sin ^2 \theta _{13} , \delta )$ and $(\sin ^2 \theta _{23} , \sin ^2\theta _{13})$, given in [23], with a rather high accuracy (see [49, 50]). We construct, e.g., $\chi ^2 (\cos \delta )$ by marginalising $\chi ^2(\{x_i\})$ over the free parameters, e.g., $\sin ^2 \theta _{13}$ and $\sin ^2 \theta _{23}$, for a fixed value of $\cos \delta$. Given the global fit results, the likelihood function,

114 $$\begin{aligned} L(\cos \delta ) \propto \exp \left( - \frac{\chi ^2(\cos \delta )}{2} \right) , \end{aligned}$$

represents the most probable values of $\cos \delta$ in each considered case. When we present the likelihood function versus $\cos \delta$ within the Gaussian approximation we use $\chi ^2_\mathrm{G} = \sum _i (y_i - \overline{y}_i)^2 / \sigma ^2_{y_i}$, with $y_i = \{\sin ^2 \theta _{12},\sin ^2 \theta _{13},\sin ^2 \theta _{23}\}$, $\overline{y}_i$ are the potential best fit values of the indicated mixing parameters and $\sigma _{y_i}$ are the prospective $1\sigma$ uncertainties in the determination of these mixing parameters. More specifically, we use as $1\sigma$ uncertainties (i) 0.7 % for $\sin ^2 \theta _{12}$, which is the prospective sensitivity of the JUNO experiment [69], (ii) 5 % for $\sin ^2 \theta _{23}$, obtained from the prospective uncertainty of 2 % [4] on $\sin ^2 2 \theta _{23}$ expected to be reached in the NOvA and T2K experiments, and (iii) 3 % for $\sin ^2 \theta _{13}$, deduced from the error of 3 % on $\sin ^2 2 \theta _{13}$ planned to be reached in the Daya Bay experiment [4, 71].

## Appendix B: $\sin ^2 \theta _{23}$ in the $(\theta ^e_{13},\theta ^e_{23})-(\theta ^\nu _{23}, \theta ^\nu _{12})$ set-up

For completeness in Fig. 8 we give $N_{\sigma } \equiv \sqrt{\chi ^2}$ as a function of $\sin ^2 \theta _{23}$ for the scheme $(\theta ^e_{13},\theta ^e_{23}) - (\theta ^\nu _{23}, \theta ^\nu _{12})$.

The dashed lines represent the results of the global fit [23], while the solid ones represent the results we obtain for each of the considered symmetry forms of the matrix $\tilde{U}_{\nu }$, minimising the value of $\chi ^2$ for a fixed value of $\sin ^2 \theta _{23}$. The blue lines correspond to the NO neutrino mass spectrum, while the red ones are for the IO one.

## Footnotes

1 If the charged lepton mass term is written in the right–left convention, the matrix $U_e$ diagonalises the hermitian matrix $M^\dagger _E M_E$, $U_e^{\dagger } M^\dagger _E M_E U_e = {{{\mathrm{diag}}}}(m^2_e,m^2_{\mu },m^2_{\tau })$, $M_E$ being the charged lepton mass matrix.

2 The phases in the matrix $Q_0$ contribute to the Majorana phases in the PMNS matrix [22].

3 Similar results were obtained in the global analysis of the neutrino oscillation data performed in [24].

4 The case of $\tilde{U}_e$ representing a rotation in the 2–3 plane is ruled out for the five symmetry forms of $\tilde{U}_{\nu }$ listed above, since in this case a realistic value of $\theta _{13} \ne 0$ cannot be generated.

5 We would like to note that the recent statistical analyses performed in [23, 24] showed indications/hints that $\delta \cong 3\pi /2$. As for $\sin ^2\theta _{12}$, $\sin ^2\theta _{13}$ and $\sin ^2\theta _{23}$, in the case of $\delta$ we utilise as “data” the results obtained in Ref. [23].

6 We consider only the “standard” ordering of the two rotations in $\tilde{U}_e$; see [46]. The case with $\tilde{U}_e = R^{-1}_{23}(\theta ^e_{23}) \, R^{-1}_{12}(\theta ^e_{12})$ has been analysed in detail in [45, 46, 49, 50] and will not be discussed by us.

7 We note that the expression for $\cos \omega$ we have obtained coincides with that for $\cos \phi$ in the set-up with the $(ij)= (12)$ rotation in the charged lepton sector (cf. Eq. (46) in [45]).

8 We note that the expression (42) for $\cos \alpha$ can be obtained formally from the r.h.s. of Eq. (22) for $\cos \phi$ in [45] by substituting $\sin \theta _{23}$ with $\cos \theta _{23}$ and vice versa and by changing its overall sign.

9 For $\sin ^2 \theta _{23} < 0.48802$, $\cos \delta$ has an unphysical (complex) value.

10 The corresponding results for IO neutrino mass spectrum differ little from those shown in the left panel of Fig. 1.

11 This sensitivity is planned to be achieved in future neutrino facilities [70].
